# Bap1/SMN axis in Dpp4^+^ skeletal muscle mesenchymal cells regulates the neuromuscular system

**DOI:** 10.1172/jci.insight.158380

**Published:** 2022-05-23

**Authors:** Ji-Hoon Kim, Jong-Seol Kang, Kyusang Yoo, Jinguk Jeong, Inkuk Park, Jong Ho Park, Joonwoo Rhee, Shin Jeon, Young-Woo Jo, Sang-Hyeon Hann, Minji Seo, Seungtae Moon, Soo-Jong Um, Rho Hyun Seong, Young-Yun Kong

**Affiliations:** 1 School of Biological Sciences, Seoul National University, Seoul, South Korea.; 2 Molecular Recognition Research Center, Korea Institute of Science and Technology, Seoul, South Korea.; 3 Institute of Molecular Biology and Genetics, Seoul National University, Seoul, South Korea.; 4 Department of Integrative Bioscience and Biotechnology, Sejong University, Seoul, South Korea.

**Keywords:** Cell Biology, Muscle Biology, Mouse models, Muscle, Neurodegeneration

## Abstract

The survival of motor neuron (SMN) protein is a major component of the pre-mRNA splicing machinery and is required for RNA metabolism. Although *SMN* has been considered a fundamental gene for the central nervous system, due to its relationship with neuromuscular diseases, such as spinal muscular atrophy, recent studies have also revealed the requirement of SMN in non-neuronal cells in the peripheral regions. Here, we report that the fibro-adipogenic progenitor subpopulation expressing Dpp4 (Dpp4^+^ FAPs) is required for the neuromuscular system. Furthermore, we also reveal that BRCA1-associated protein-1 (Bap1) is crucial for the stabilization of SMN in FAPs by preventing its ubiquitination-dependent degradation. Inactivation of *Bap1* in FAPs decreased SMN levels and accompanied degeneration of the neuromuscular junction, leading to loss of motor neurons and muscle atrophy. Overexpression of the ubiquitination-resistant SMN variant, *SMN^K186R^*, in *Bap1*-null FAPs completely prevented neuromuscular degeneration. In addition, transplantation of Dpp4^+^ FAPs, but not Dpp4^–^ FAPs, completely rescued neuromuscular defects. Our data reveal the crucial role of *Bap1*-mediated SMN stabilization in Dpp4^+^ FAPs for the neuromuscular system and provide the possibility of cell-based therapeutics to treat neuromuscular diseases.

## Graphical abstract

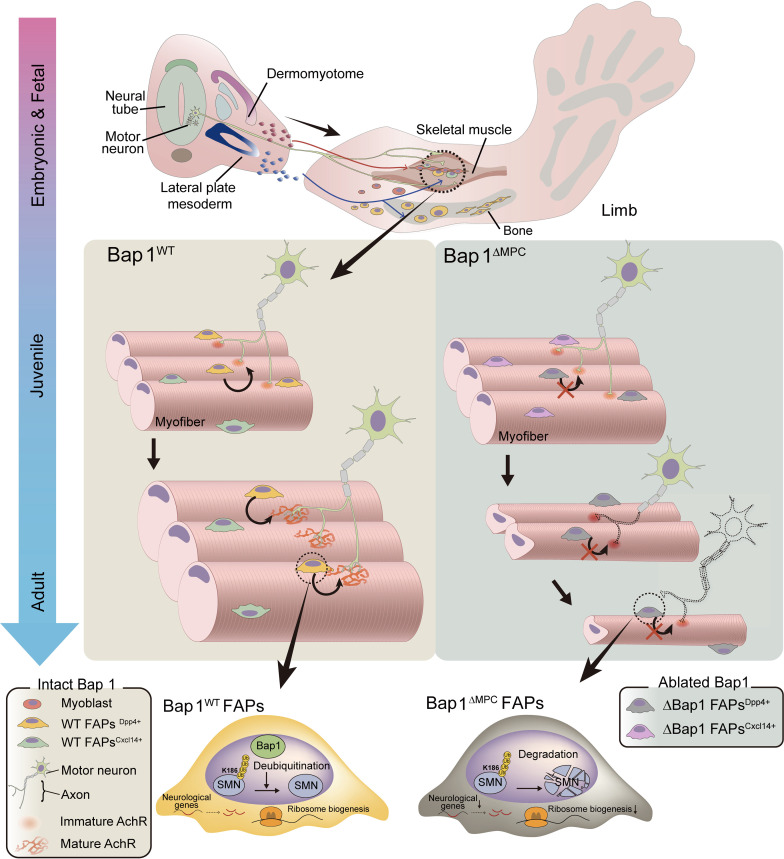

## Introduction

Spinal muscular atrophy (SMA) is an autosomal-recessive neurodegenerative disease caused by mutations in the survival of motor neuron 1 (*SMN1*) gene (1). The primary clinical manifestation of this disease is the functional loss of lower motor neurons. Thus, SMA had been regarded as a motor neuron disease caused by insufficient SMN in motor neurons to retain their function and survival. However, motor neuron–specific SMN depletion is not as deleterious to motor neurons and motor function as ubiquitously depleting SMN (2). Conversely, selective SMN restoration in the central nervous system (CNS) and motor neuron–specific delivery of SMN show only partial rescue effects in SMA mouse models (3–6). In addition, peripheral restoration of SMN by an antisense oligonucleotide, which corrects *SMN2* splicing, was able to rescue motor function and motor neuron loss in SMA mouse models (7, 8). These results suggest an indispensable role of non-neuronal cells in the neuromuscular system. Recent studies reported SMN requirements in neuromuscular junction (NMJ) components, such as Schwann cells (9–11), and these results imply relations between insufficient SMN, NMJ defects, and SMA pathogenesis. Notwithstanding accumulating evidence, non-neuronal peripheral cells that require SMN to protect motor neurons from degeneration have not been fully identified.

SMN is ubiquitously expressed, but its expression level varies depending on the developmental stages or the types of tissue. For instance, there is a general decrease in SMN level during the postnatal growth period, while it remains higher in the central nervous tissues than in the peripheral tissues (12, 13). Furthermore, SMN protein levels do not linearly correlate with its transcript levels in many cases (13, 14), indicating distinct posttranscriptional regulatory mechanisms in different cell types. The ubiquitin-proteasome system (UPS) is the most studied posttranslational mechanism that regulates SMN levels, in which several E3 ubiquitin ligases (e.g., Mib1, Itch, UCHL1) and the deubiquitinase (DUB) Usp9x have been suggested (15–18). However, regulation of SMN by UPS was mostly studied in in vitro systems or *Caenorhabditis* *elegans* or *Drosophila* models, meaning that the direct relevance of UPS-dependent SMN regulation to SMA in mammalian tissues is not fully elucidated. Thus, investigating the posttranslational regulation of SMN in various mammalian tissues that are responsible for SMA is fundamental to understanding SMA pathogenesis.

Mesenchymal progenitor cells (MPCs) in skeletal muscle originate from the somatic lateral plate mesoderm and, after migrating into the limb buds (19, 20), give rise to the bone, cartilage, and connective tissues. During developmental myogenesis, odd-skipped related transcription factor 1–positive MPCs support muscle patterning by providing promyogenic niches (21). Some of the MPCs intermingle with muscle progenitor cells derived from the abaxial dermomyotome and become skeletal muscle interstitial mesenchymal cells residing between muscle fibers (20, 22). Skeletal muscle mesenchymal cells are also known as fibro-adipogenic progenitors (FAPs) because of their in vitro differentiation potential (23). During muscle and nerve injury, FAPs support muscle regeneration and sense denervation that leads to muscle atrophy, respectively (24–27). Previous research mainly focused on the role of FAPs in injured rather than homeostatic muscles. However, recent studies revealed the relevance of FAPs to diseases, such as muscular atrophy or sarcopenia, suggesting an essential role of FAPs in muscle homeostasis during adulthood (27–29). Furthermore, recent studies revealed that FAPs are a heterogeneous cell population, which consists of 2 subpopulations: FAP1 and FAP2 (30, 31). Although FAP1 and FAP2 show different gene expression characteristics, suggesting their distinct roles, the exact function of each subpopulation remains largely unknown.

BRCA1-associated protein-1 (*Bap1*), also known as ubiquitin carboxyl-terminal hydrolase 2, is a deubiquitinating enzyme. It plays a role in DNA damage, cell cycle, and tumorigenesis by regulating the stability of various substrates and the amount of ubiquitinated histone H2A (32–34). Since *Bap1*-knockout mice are early embryonic lethal (35), the physiological role of *Bap1* in postnatal development remains largely unknown. In this study, we aimed to investigate the physiological function of *Bap1* in MPCs in vivo by generating *Bap1*^fl/fl^ mice and inactivating the *Bap1* gene in MPCs, including FAPs. Our study revealed that Bap1 regulated SMN levels in FAPs and that the *Dpp4*^+^ FAP subpopulation had a crucial role in the neuromuscular system, distinct from the other FAP subpopulation. These results provided valuable insights into UPS-dependent regulation of SMN by Bap1 in FAPs, the physiological function of FAPs in muscle homeostasis, SMA pathogenesis, and treatment of SMA.

## Results

### Severe muscular atrophy in Bap1^ΔMPC^ mice.

**Figure 1:**
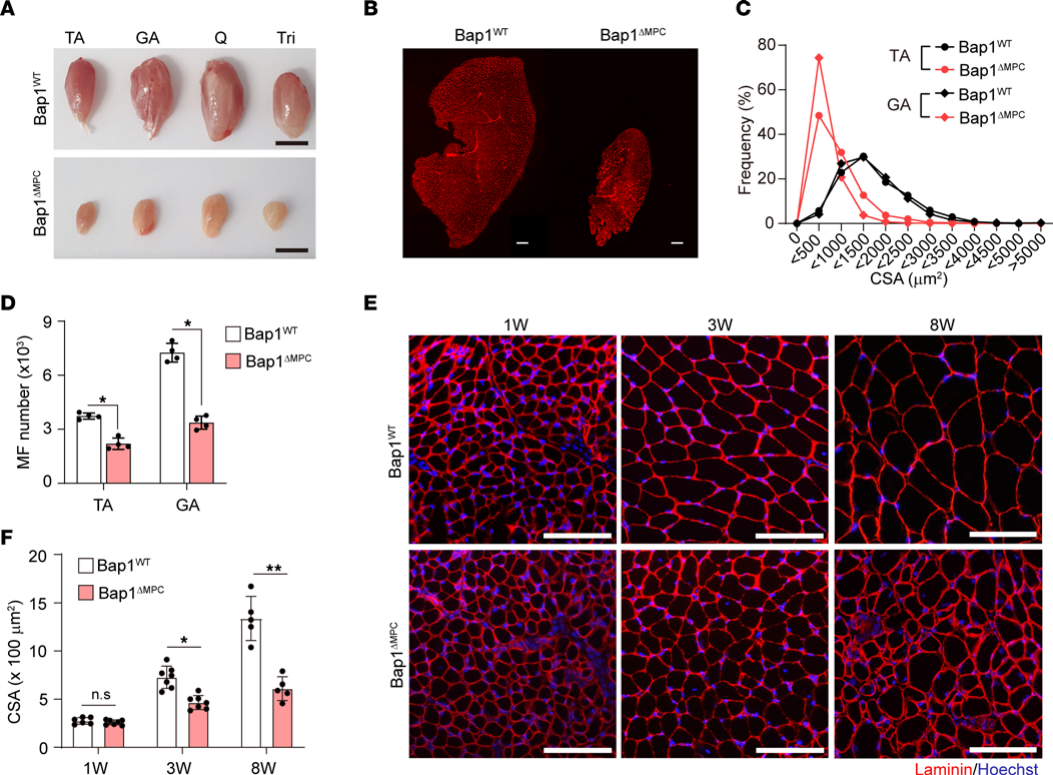
Severe muscular atrophy in *Bap1*^ΔMPC^ mice. (**A**) Gross morphology of tibialis anterior (TA), gastrocnemius (GA), quadriceps (Q), and triceps (Tri) muscles from 19-month-old *Bap1*^WT^ and *Bap1*^ΔMPC^ mice. (**B**) Representative IHC staining images of laminin. (**C**) Morphometric quantifications of CSA of TA and GA myofibers from *Bap1*^WT^ and *Bap1*^ΔMPC^ mice. (**D**) Quantification of myofiber numbers in whole TA and GA. (**E**) Representative IHC staining images of TA muscles of 1-, 3-, and 8-week-old *Bap1*^WT^ or *Bap1*^ΔMPC^ mice. (**F**) Morphometric quantifications of CSA of TA muscles. *n* = 4 animals per group; mean ± SEM; Mann-Whitney *U* test; *P < 0.05, **P < 0.01. Scale bars: 100 μm (**A**, **B**, and **E**).

To investigate the physiological function of Bap1 in MPCs of skeletal muscle, we generated *Bap1*^fl/fl^ mice (Supplemental Figure 1A; supplemental material available online with this article; https://doi.org/10.1172/jci.insight.158380DS1) and inactivated the *Bap1* gene by crossing *Bap1*^fl/fl^ mice with *Prx1-Cre* transgenic mice (*Prx1-Cre* *Bap1*^fl/fl^, hereafter *Bap1*^ΔMPC^). *Bap1* was significantly disrupted in FAPs, the MPC-derived cells in skeletal muscle, but not in other cell populations, such as Schwann cells or muscle stem cells (Supplemental Figure 1, B–D). Since *Bap1* has been implicated in tumorigenesis (36), we monitored these mice for more than 18 months but found no sign of tumors in the MPC-derived tissues, including peritoneum (data not shown). Instead, *Bap1*^ΔMPC^ mice showed severely defective limb movements at 19 months of age (Supplemental Video 1). Furthermore, the masses of limb muscles were markedly decreased in *Bap1*^ΔMPC^ mice compared with those in control (*Bap1*^fl/fl^, hereafter *Bap1*^WT^) mice (Figure 1A). Histological analysis revealed drastic decreases in both cross-sectional area (CSA) and myofiber number in *Bap1*^ΔMPC^ mice (Figure 1, B–D), indicating severe muscle atrophy in *Bap1*^ΔMPC^ mice.

To determine the time point at which muscle atrophy occurs in *Bap1*^ΔMPC^ mice, we examined postnatal muscle development. Though no difference in body weight and muscle mass was observed until 1 week after birth, *Bap1*^ΔMPC^ mice showed progressively defective postnatal growth in both body weight and muscle mass normalized to body weight (Supplemental Figure 1, E and F). The TA muscle from 3-week-old *Bap1*^ΔMPC^ mice showed significantly decreased myofiber CSA compared with that of *Bap1*^WT^ mice (Figure 1, E and F). These results revealed that inactivation of *Bap1* in FAPs gives rise to severe muscular atrophy.

### Impaired motor functions in Bap1^ΔMPC^ mice.

**Figure 2:**
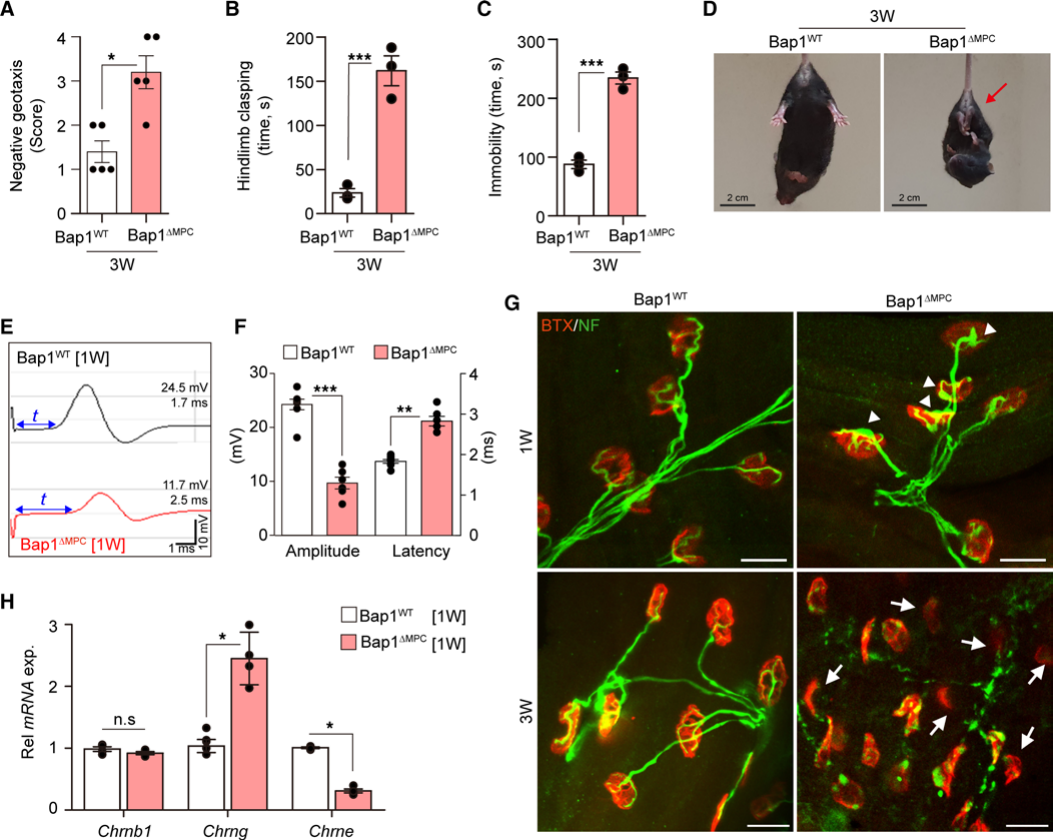
Impaired motor functions and NMJs in *Bap1*^ΔMPC^ mice. (**A**) The score of negative geotaxis test (38). (**B** and **C**) hind limb clasping time (85) (**B**) and immobility time (89) (**C**) during tail suspension test of 3-week-old *Bap1*^WT^ and *Bap1*^ΔMPC^ mice. *n* = 5 animals per group; mean ± SEM; Mann-Whitney *U* test; *P < 0.05, ***P < 0.001. (**D**) Representative captures of *Bap1*^WT^ and *Bap1*^ΔMPC^ mice during tail suspension test. Arrow indicates the hind limb clasping. (**E** and **F**) Representative graph data (**E**) and quantifications of amplitude and latency (**F**) of CMAP from 1-week-old GA muscles of *Bap1*^WT^ and *Bap1*^ΔMPC^ mice. (**G**) Confocal images for BTX and neurofilament immunofluorescence in TA muscles. White arrowheads and arrows indicate swelling of neurofilament and denervation, respectively. (**H**) Relative mRNA expressions of each AChR subunit in TA muscles. (**F** and **H**) *n* = 4 animals per group; data are mean ± SEM; Mann-Whitney *U* test; *P < 0.05, **P < 0.01, ***P < 0.001. Scale bars: 2 cm (**D**) and 20 μm (**G**).

With the muscular atrophy, *Bap1*^ΔMPC^ mice also showed apparent defects in motor functions, such as failed negative geotaxis, abnormal hind limb clasping, and immobility (Figure 2, A–D), which are predominant signs of neurological defects (37, 38). Since *Bap1*^ΔMPC^ mice had both muscular atrophy and motor functional defect phenotypes, we speculated that the neurological defect may have caused muscle atrophy or vice versa. To confirm these possibilities, we performed electromyography to measure the sciatic compound muscle action potential (CMAP), which represents the integrity of the NMJs in the lower hind limb muscles (Supplemental Figure 2A and ref. 39). We observed a decrease in amplitude and a delay in latency of CMAP in *Bap1*^ΔMPC^ mice at 1 week of age and beyond (Figure 2, E and F; and Supplemental Figure 2, B and C), which preceded the onset of muscular atrophy in *Bap1*^ΔMPC^ mice (Figure 1, E and F). Although CMAP amplitude could be affected by muscle atrophies, we construed the alteration of CMAP in our mouse model as a defective NMJ due to the absence of muscle atrophy at 1 week of age. Hence, we concluded that ablation of *Bap1* in FAPs results in NMJ dysfunction, which then leads to severe muscular atrophy in *Bap1*^ΔMPC^ mice.

### Defective neuromuscular junctions in Bap1^ΔMPC^ mice.

To investigate the cause of NMJ dysfunction in *Bap1*^ΔMPC^ mice, we examined the morphology of NMJs with rhodamine-conjugated α-bungarotoxin and anti-neurofilament antibodies to label acetylcholine receptor (AChR) and neurofilament, respectively. Intriguingly, swelling of axons in NMJs was frequently observed in 1-week-old *Bap1*^ΔMPC^ mice, while thin, elongated axons in NMJs were observed in 1-week-old *Bap1*^WT^ mice (Figure 2G). Since the defects of NMJs were observed at 1 week, when the NMJ maturation occurs, we investigated whether the switch of AChR subunits from embryonic-type γ (*Chrng*) to adult-type ε (*Chrne*) occurs during early postnatal development in *Bap1*^ΔMPC^ mice (40, 41). Expectedly, *Chrng* levels increased whereas *Chrne* levels decreased in 1-week-old *Bap1*^ΔMPC^ mice, compared with those in *Bap1*^WT^ mice (Figure 2H). Furthermore, nerve terminals showed degenerations, and NMJs were denervated in *Bap1*^ΔMPC^ mice by 3 weeks of age (Figure 2G). These results indicate that the loss of Bap1 in non-neuronal FAPs results in the failure of NMJ maturation in *Bap1*^ΔMPC^ mice.

**Figure 3:**
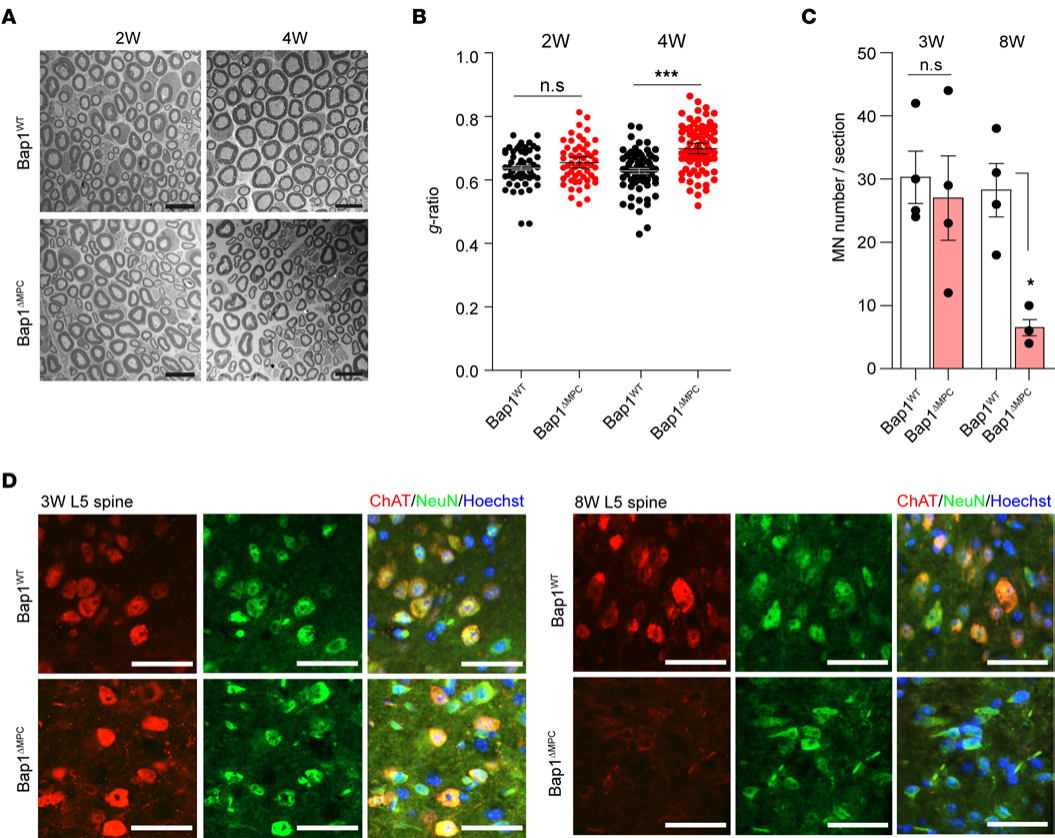
Loss of motor neurons in *Bap1*^ΔMPC^ mice. (**A**) Representative electron microscopy images of myelinated axons in sciatic nerve cross sections from 2- (left panels) or 4-week-old (right panels) *Bap1*^WT^ and *Bap1*^ΔMPC^ mice. (**B**) The g-ratio of sciatic nerves from 2- or 4-week-old *Bap1*^WT^ and *Bap1*^ΔMPC^ mice. (**C** and **D**) Quantifications of motor neuron (MN) number (**C**) and representative IHC images of choline acetyltransferase (ChAT) and NeuN in the ventral horn regions of L5 spinal cord from 3- or 8-week-old *Bap1*^WT^ and *Bap1*^ΔMPC^ mice (**D**). *n* = 4 animals for each group (**B** and **C**); data are mean ± SEM; Mann-Whitney *U* test; *P < 0.05, ***P < 0.001. Scale bars: 10 μm (**A**) and 50 μm (**D**).

To ascertain whether the defects in NMJs lead to motor neuron abnormalities, we examined axon size and myelination thickness of sciatic nerves. There was no difference between *Bap1*^WT^ and *Bap1*^ΔMPC^ mice in the axon size and the myelination thickness at 2 weeks of age. By 4 weeks of age, however, *Bap1*^ΔMPC^ mice showed a significant decrease in both features compared with *Bap1*^WT^ mice (Figure 3, A and B). Furthermore, we examined the cell body of motor neurons at the ventral horn of L5 spinal cord and observed that the number of motor neurons was markedly decreased in *Bap1*^ΔMPC^ mice at 8 weeks of age, but not at 3 weeks of age (Figure 3, C and D). Taken together, our observations revealed that the defective NMJ maturation accompanies not only muscle atrophy but also the progressive loss of motor neurons in *Bap1*^ΔMPC^ mice. Furthermore, these results implied that FAPs in skeletal muscles, targeted by Prx1-Cre in our *Bap1*^ΔMPC^ mice (Supplemental Figure 1, B–D), could play a critical role in the establishment of the neuromuscular system.

### Prevention of neuromuscular degeneration by FAPs.

**Figure 4:**
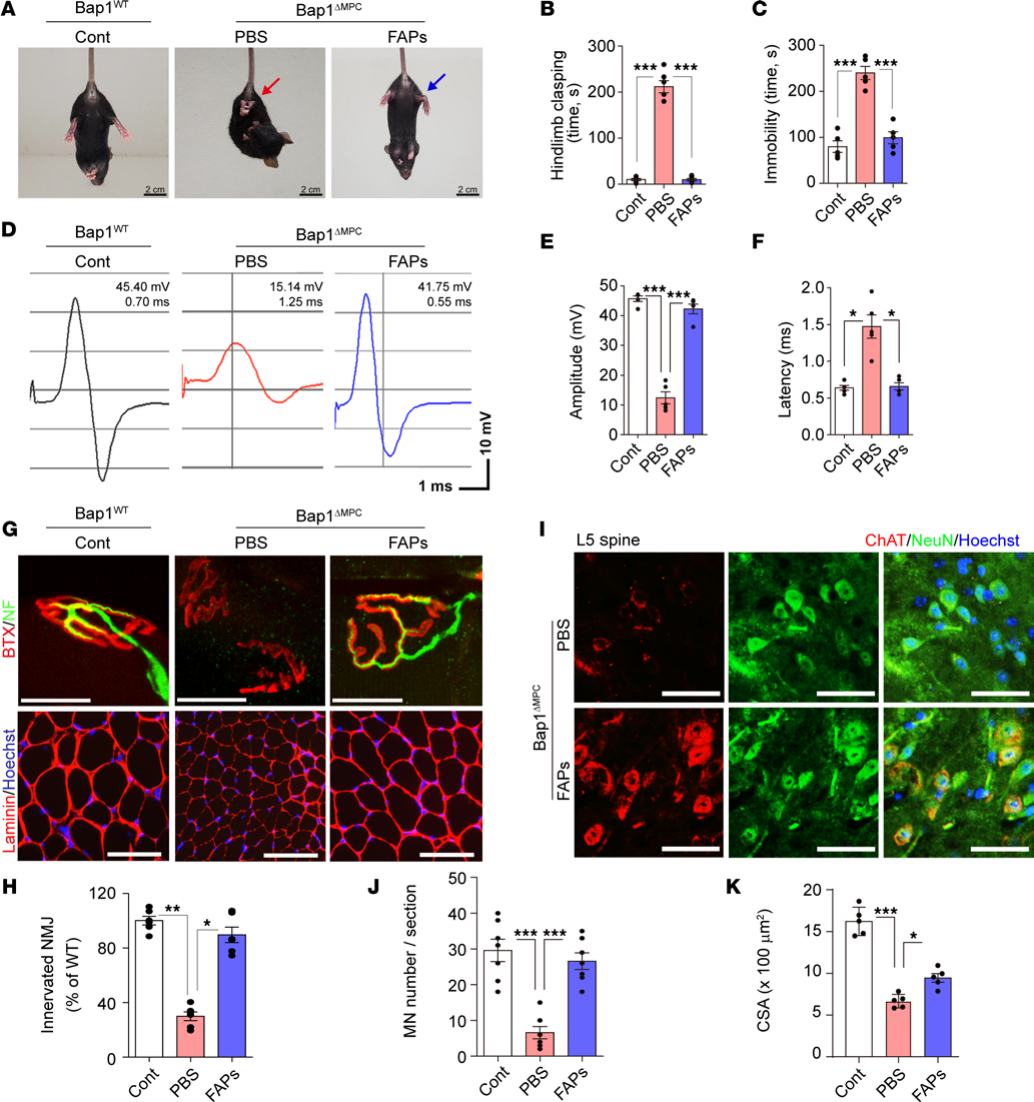
Prevention of neuromuscular degeneration by FAPs. (**A**) Representative captures from tail suspension test of *Bap1*^WT^ (Cont), PBS-treated (PBS), or FAP-transplanted (FAPs) *Bap1*^ΔMPC^ mice. See also Supplemental Video 2. (**B** and **C**) Quantifications of hind limb clasping time (**B**) and immobility time (**C**) during tail suspension test for 5 minutes. (**D**) Representative graph data of CMAP. (**E** and **F**) Quantifications of amplitude (**E**) and latency (**F**) of CMAP measured in GA muscles of Cont, PBS, and FAPs mice. (**G**) Confocal images of BTX and NF immunofluorescence (top) and IHC images of TA muscles (bottom). (**H**) Percentages of the innervated NMJs. (**I** and **J**) Representative IHC images of ChAT and neurofilament (NF) in ventral horn regions of L5 spinal cord (**I**) and quantifications of MN number (**J**). (**K**) Quantification of CSA. *n* = 5 animals for each group (**B**, **C**, **E**, **F**, **H**, **J**, and **K**) (*n* = 6 animals in **J**); data are mean ± SEM; Tukey’s pairwise comparison test after 1-way ANOVA; *P < 0.05, **P < 0.01, ***P < 0.001. Scale bars: 2 cm (**A**), 20 μm (**G**, top), 100 μm (**G**, bottom), and 50 μm (**I**).

FAPs are the only cell population derived from the somatic lateral plate mesoderm among cellular components in limb skeletal muscles (20, 42). For confirmation, we crossed the *Rosa-YFP* reporter mouse with the *Prx1-Cre* mouse (hereafter, *Prx1-Cre* *Rosa-YFP*) and observed that only FAPs were YFP positive (Supplemental Figure 3A). Thus, to investigate whether FAPs indeed play a critical role in the establishment of the neuromuscular system, we transplanted freshly isolated WT YFP^+^ FAPs from *Prx1-Cre* *Rosa-YFP* mice into hind limb muscles of 1.5-week-old *Bap1*^ΔMPC^ mice (Supplemental Figure 3B). Interestingly, 8 weeks after transplantation, FAP-transplanted *Bap1*^ΔMPC^ mice showed dramatically improved motor functions in tail suspension tests compared with PBS-treated *Bap1*^ΔMPC^ mice (Figure 4, A–C, and Supplemental Video 2). Moreover, the decreased amplitude and the delayed latency of CMAP were also completely improved up to the levels observed in *Bap1*^WT^ mice (Figure 4, D–F).

To investigate whether WT FAP transplantation can also rescue the defective NMJ maturation in *Bap1*^ΔMPC^ mice, we examined the structure of NMJs in *Bap1*^ΔMPC^ mice 8 weeks after WT FAP transplantation. PBS-treated *Bap1*^ΔMPC^ mice showed features of defective NMJs, such as fragmented AChR clusters and regressed neurofilaments. However, FAP-transplanted *Bap1*^ΔMPC^ mice had intact AChR clusters with multiperforated, pretzel-like structures, and the percentage of innervation was comparable to *Bap1*^WT^ mice (Figure 4, G and H). Moreover, while PBS-treated *Bap1*^ΔMPC^ mice also showed a significant decrease in motor neuron number in the ventral horn of L5 spinal cord, FAP-transplanted *Bap1*^ΔMPC^ mice had similar numbers of motor neurons with *Bap1*^WT^ mice (Figure 4, I and J). These results indicate that the transplantation of WT FAPs rescues defective NMJ maturation and prevents motor neuron loss in *Bap1*^ΔMPC^ mice.

Furthermore, histological analyses revealed a considerable increase in myofiber size by FAP transplantation (Figure 4, G and K, and Supplemental Figure 3C). These results showed that transplantation of FAPs completely prevented the degeneration of the neuromuscular system in *Bap1*^ΔMPC^ mice. Collectively, we concluded that non-neuronal FAPs play a critical role in the maturation of NMJ and the protection of muscular atrophy and motor neuron degeneration.

### Maintenance of the neuromuscular system by FAPs.

**Figure 5:**
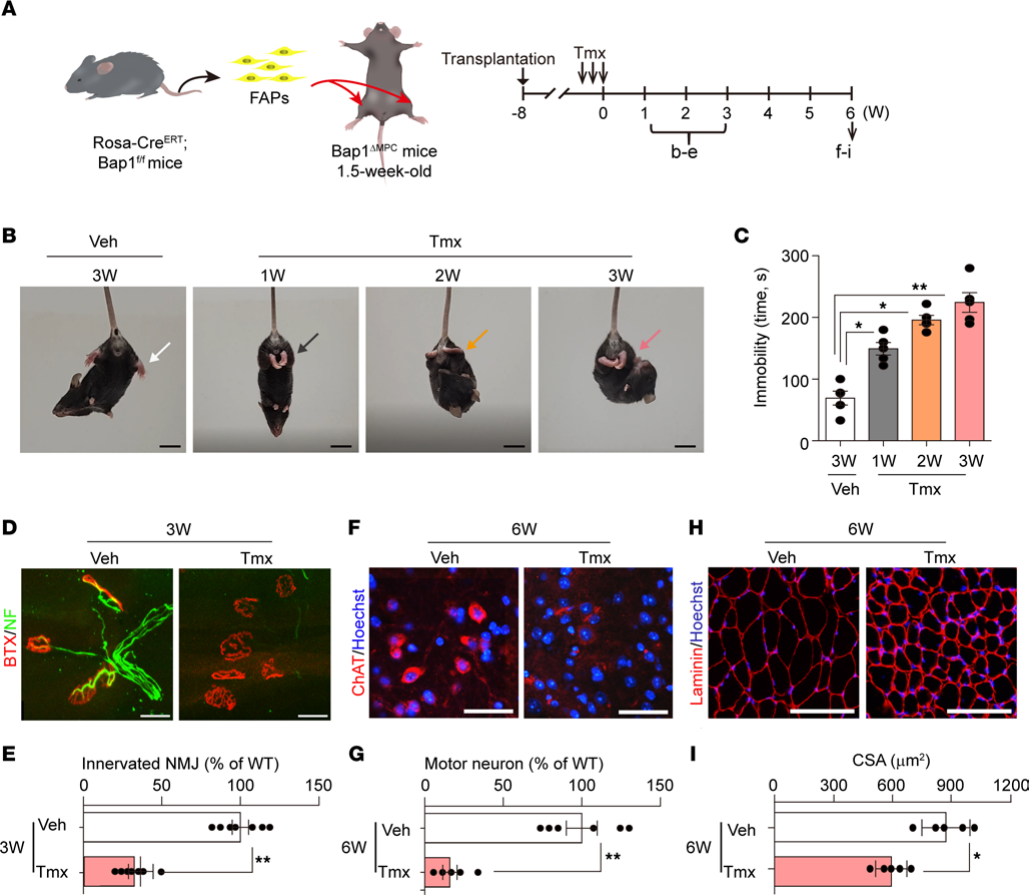
Maintenance of the neuromuscular system by FAPs. (**A**) Experimental scheme of FAPs’ transplantation. FAPs from hind limb muscles of 1.5-week-old *Rosa-CreER* *Bap1*^fl/fl^ mice were transplanted into TA and GA muscles of 1.5-week-old *Bap1*^ΔMPC^ mice. Eight weeks after transplantation, the transplanted mice were orally administered with tamoxifen (Tmx) for 3 consecutive days. (**B** and **C**) Tail suspension test was performed at the indicated weeks after Tmx administration. Representative captures of tail suspension tests (**B**). Arrow indicates the hind limb clasping. See also Supplemental Video 3. Quantification of immobility time during tail suspension test for 5 minutes (**C**). *n* = 5 animals for each group; data are mean ± SEM; Tukey’s pairwise comparison test after 1-way ANOVA; *P < 0.05, **P < 0.01. (**D**–**G**) Histological analyses were performed at the indicated weeks after Tmx administration. Representative confocal images of NMJs (**D**) and quantification of innervated NMJs (**E**). Representative IHC images of ChAT (**F**) and quantifications of MNs (**G**) in L5 spinal cords. (**H**) Representative IHC staining images of TA muscles. (**I**) Quantification of CSA in TA muscles. (**E**, **G**, and **I**) *n* = 5 animals for each group; data are mean ± SEM; unpaired *t* test; **P < 0.01, ***P < 0.001. Scale bars: 2 cm (**B**), 25 μm (**D**), 50 μm (**F**), and 100 μm (**H**).

To investigate whether FAPs are also required for the maintenance of the neuromuscular system in adulthood, we isolated FAPs from *Bap1*^fl/fl^ mice crossed with *Rosa-CreER* transgenic mice (hereafter, *Rosa-CreER* *Bap1*^fl/fl^ FAPs; ref. 43) and transplanted them into 1.5-week-old *Bap1*^ΔMPC^ mice (Figure 5A). In this case, the transplanted cells are the only FAPs expressing *Bap1* in the mutant mouse. Then, we administered tamoxifen to disrupt the *Bap1* gene in the transplanted FAPs 8 weeks posttransplantation, when sufficient rescue effects were observed. Surprisingly, 3 weeks after tamoxifen administration, the cell-transplanted *Bap1*^ΔMPC^ mice showed abnormal hind limb clasping and immobility during tail suspension tests (Figure 5, B and C, and Supplemental Video 3). To investigate whether these motor function defects are due to failure of NMJ maintenance, we examined NMJs in TA muscles of cell-transplanted, tamoxifen-administered *Bap1*^ΔMPC^ mice. Consistent with the motor function defects, most NMJs were denervated at 3 weeks posttamoxifen administration, while innervation of NMJs was maintained in TA muscles of the vehicle-treated, cell-transplanted *Bap1*^ΔMPC^ mice (Figure 5, D and E). Moreover, 6 weeks after tamoxifen administration, NMJ denervation gave rise to the loss of motor neurons in L5 spinal cord (Figure 5, F and G) and decrease in CSA (Figure 5, H and I). Collectively, these results indicate that the non-neuronal FAPs are also required to maintain the neuromuscular system throughout life.

### Prevention of neuromuscular degeneration by Dpp4^+^-FAPs.

**Figure 6:**
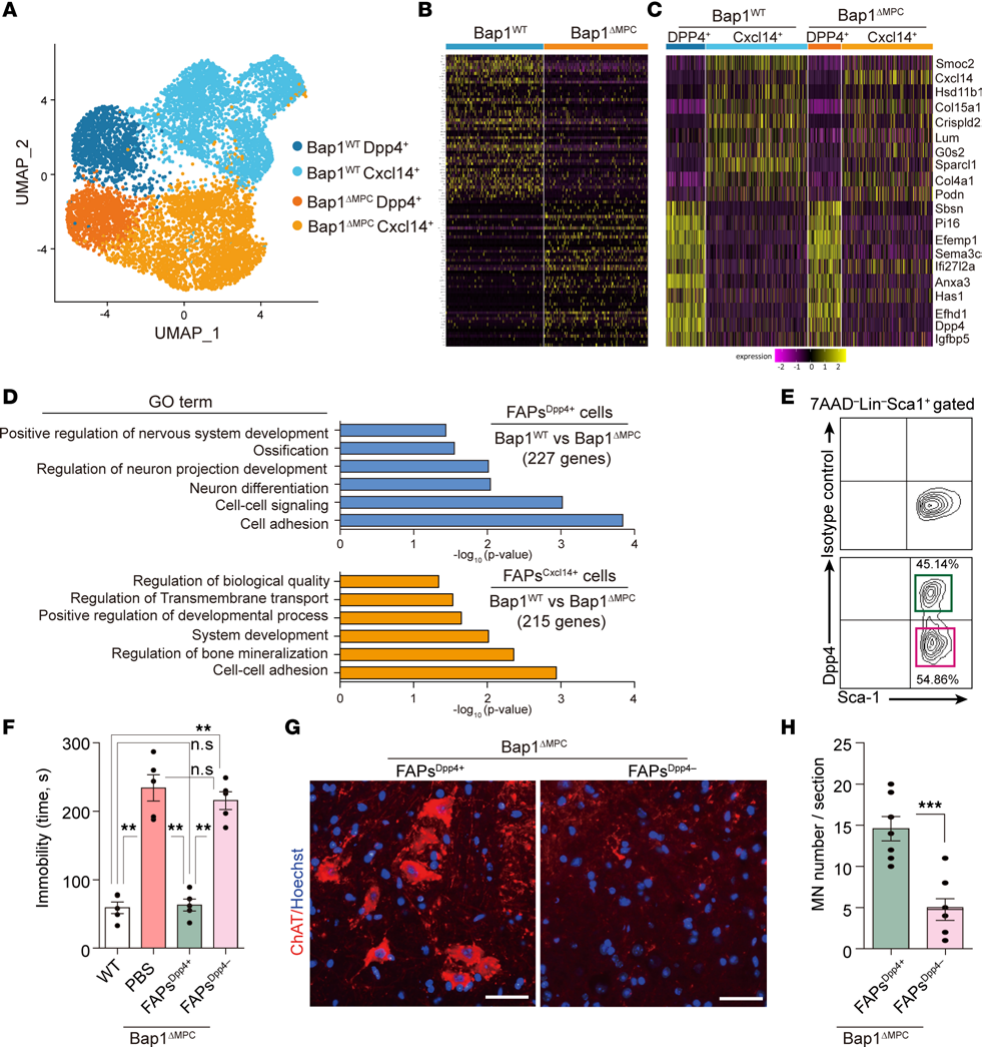
Identification of the neuroprotective Dpp4^+^ subpopulation within FAPs. (**A**) Uniform manifold approximation and projection (UMAP) plot showing distinct subpopulations (Dpp4^+^ or Cxcl14^+^) within *Bap1*^WT^ versus *Bap1*^ΔMPC^ FAPs. (**B**) Heatmap of top 100 differentially expressed genes in *Bap1*^WT^ versus *Bap1*^ΔMPC^ FAPs. (**C**) Heatmap showing expression of marker genes used to distinguish the 2 subpopulations in each cluster. (**D**) GO analysis using statistical overrepresentation test on DEGs selected from comparing *Bap1*^WT^ versus *Bap1*^ΔMPC^ FAPs in each subpopulation. (**E**) Representative FACS plots of Dpp4^+^ versus Dpp4^–^ FAPs isolated from 4-week-old tdTomato reporter mice. (**F**) Quantification of immobility time during tail suspension test. *Bap1*^ΔMPC^ mice transplanted with PBS, Dpp4^+^ FAPs, or Dpp4^–^ FAPs were compared with *Bap1*^WT^ mice. *n* = 5 animals for each group; data are mean ± SEM; Tukey’s pairwise comparison test after 1-way ANOVA; **P < 0.01. (**G** and **H**) Representative IHC images of ChAT (scale bars: 50 μm) (**G**) and quantifications of MN number stained with ChAT on the ventral horn region of L5 spinal cord in Dpp4^+^ FAP– or Dpp4^–^ FAP–transplanted *Bap1*^ΔMPC^ mice (**H**). *n* = 5 animals for each group. Mean ± SEM; unpaired *t* test; ***P < 0.001.

Single-cell RNA-sequencing (scRNA-Seq) experiments from previous studies revealed that the FAPs consist of 2 subpopulations: FAP1 and FAP2, expressing Cxcl14 and Dpp4 as their marker genes, respectively (30, 31, 44). Although gene expression profiles between FAP1 (hereafter, Cxcl14^+^ FAPs) and FAP2 (hereafter, Dpp4^+^ FAPs) suggested their distinctive roles in muscle physiology, the different functions of each subpopulation have not been investigated to our knowledge. Thus, to determine the FAP subpopulation implicated in neuromuscular function, we subjected FAPs from 3-week-old *Bap1*^WT^ and *Bap1*^ΔMPC^ mice to scRNA-Seq analysis, a time point when the neuromuscular phenotypes were apparent (Supplemental Figure 4A). As a result, unsupervised clustering of FAPs from *Bap1^WT^* versus *Bap1*^ΔMPC^ mice revealed apparent differences in their transcriptomic profiles (Figure 6, A and B, and Supplemental Figure 4B). Also, FAPs from both genotypes were readily divided into Dpp4^+^/Cxcl14^+^ FAPs by the expression of marker genes previously reported (Figure 6C and Supplemental Figure 4C). The 4 clusters of cells also showed unique expression of signature genes, further suggesting their distinct effects on muscle physiology (Supplemental Figure 4, D and E, and Supplemental Figure 5A). To infer the physiological relevance of *Bap1* ablation in each subpopulation, we identified differentially expressed genes (DEGs) in each subpopulation (Supplemental Figure 5B) and subjected them to gene ontology (GO) enrichment analysis. Surprisingly, *Bap1* ablation resulted in transcriptomic changes related to neurological functions specifically in Dpp4^+^ FAPs but not in Cxcl14^+^ FAPs (Figure 6D), implying that Dpp4^+^ FAPs are responsible for neuromuscular function. To verify this possibility, we isolated Dpp4^+^ and Dpp4^–^ FAPs by FACS from 4-week-old tdTomato reporter mice and transplanted them into *Bap1*^ΔMPC^ mice (Figure 6E and Supplemental Figure 6A). RNA expressions of marker genes in the FACS-sorted cells confirmed a clear separation of Dpp4^+^ FAPs from Dpp4^–^ (Cxcl14^+^) FAPs (Supplemental Figure 5, C and D). Intriguingly, Dpp4^+^ FAPs, but not Dpp4^–^ FAPs, were able to rescue the neuromuscular defects in *Bap1*^ΔMPC^ mice (Figure 6, F–H; Supplemental Figure 6, B–H; and Supplemental Video 4), showing that Dpp4^+^ FAPs are the bona fide FAP subpopulation required for the establishment and maintenance of the neuromuscular system.

### Deubiquitination of SMN by Bap1.

**Figure 7:**
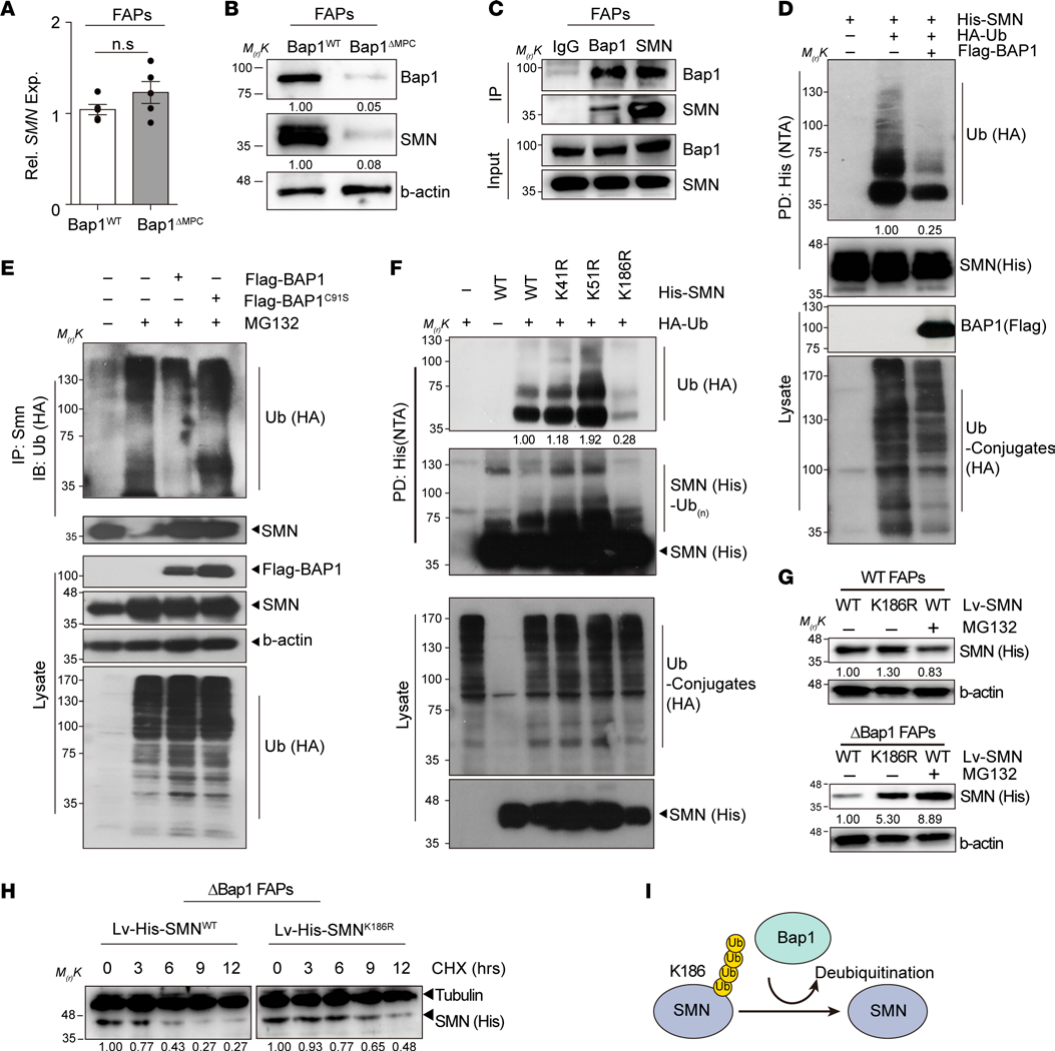
Deubiquitination of SMN by Bap1. (**A**) Relative expression of *SMN* in FAPs from hind limb muscles of 1.5-week-old *Bap1*^WT^ and *Bap1*^ΔMPC^ mice. *Gapdh* and *b-actin* were used for normalization in quantitative PCR analysis. *n* = 4 animals for each group; Mann-Whitney *U* test; data are mean ± SEM. (**B**) Immunoblot of Bap1 and SMN in FAPs from 4-week-old *Bap1*^WT^ and *Bap1*^ΔMPC^ mice. (**C**) Immunoblot of Bap1 and SMN after immunoprecipitation (IP) analysis. IP was performed with pre-immune IgG, anti-Bap1, or anti-SMN antibodies. Each sample was blotted with each indicated antibody. (**D**) Ubiquitination assay of SMN following BAP1 overexpression. Flag-tagged BAP1 was expressed in HEK293T cells with HA-Ubiquitin (HA-Ub) and HisMax-tagged SMN (His-SMN). Cell lysates were subjected to pull-down assays with NTA resins followed by immunoblot. (**E**) Ubiquitination assay of SMN following BAP1 or BAP1^C91S^ overexpression. HEK293T cells expressing Flag-tagged BAP1 or its C91S mutant were subjected to IP with anti-SMN antibody. After IP, each sample was blotted with each indicated antibody. (**F**) Ubiquitination assay of SMN and its lysine-substitution variants. HisMax-tagged variants (K41R, K51R, or K186R) were expressed in HEK293T cells with or without HA-Ub. Cell lysates were subjected to pull-down assays with NTA resins followed by immunoblot with each indicated antibody. (**G**) Immunoblot of SMN in *Bap1*^WT^ or *Bap1*^ΔMPC^ FAPs transduced with lentiviral vectors containing His-tagged SMN^WT^ or SMN^K186R^. Samples were blotted with each indicated antibody. (**H**) Immunoblot analysis of SMN protein half-life. *Bap1*^ΔMPC^ FAPs transduced with lentiviral vectors containing His-tagged SMN or its K186R mutant were subjected to immunoblot with indicated antibodies. Cells were incubated with cycloheximide (CHX) for the indicated times. (**I**) A schematic model for deubiquitination of SMN1 by BAP1. (**D**, **F**, and **G**) For MG132 treatment, cells were incubated with 10 μM MG132 for 4 hours before the preparation of cell lysates.

To gain insight into the mechanism by which Bap1 regulates the function of FAPs, we performed RNA-Seq analysis using freshly isolated FAPs from hind limb skeletal muscles of 1.5-week-old *Bap1*^WT^ and *Bap1*^ΔMPC^ mice. Interestingly, *Bap1*^ΔMPC^ FAPs showed decreased expressions of genes categorized into RNA processing by GO enrichment analysis, compared with *Bap1*^WT^ FAPs (Supplemental Figure 7, A–D). The defective RNA processing and ribonucleoprotein complex biogenesis, together with the motor function defects, are representative phenotypes of SMA, a neuromuscular disease caused by insufficient SMN protein level (12, 45–47). Thus, we anticipated the alteration of SMN in *Bap1*^ΔMPC^ FAPs and compared SMN levels in FAPs from *Bap1*^WT^ and *Bap1*^ΔMPC^ mice. Indeed, the protein level of SMN was drastically decreased in *Bap1*^ΔMPC^ FAPs compared with *Bap1*^WT^ FAPs, while there was no difference in mRNA level (Figure 7, A and B). Furthermore, in line with the reduced SMN protein level, we observed a decrease in levels of small nuclear RNAs (snRNAs) in *Bap1*^ΔMPC^ FAPs as has been reported in SMA mice (48, 49) (Supplemental Figure 7E). These results indicate that Bap1 posttranscriptionally regulates SMN levels and thus affects the function of SMN in FAPs.

To test the direct interaction between Bap1 and SMN, we performed endogenous reciprocal immunoprecipitation experiments using freshly isolated FAPs and revealed that Bap1 directly binds SMN in FAPs (Figure 7C). Semi-endogenous co-immunoprecipitation of SMN with intact or truncated Bap1 revealed that the ubiquitin C-terminal hydrolase domain of Bap1 is essential for the binding of Bap1 to SMN (Supplemental Figure 7F). Next, we investigated the ubiquitination state of SMN depending on Bap1, since Bap1 is known as a deubiquitinating enzyme and UPS is the most common pathway of posttranscriptional modifications. Notably, overexpression of Bap1 reversed the ubiquitination of SMN (Figure 7D), and this result indicated that Bap1 could regulate SMN levels by acting as a deubiquitinating enzyme. To further confirm the deubiquitinating function of Bap1 on SMN, we performed a loss-of-function experiment by introducing a nonfunctional mutation in the catalytic domain of Bap1 (*Bap1*^C91S^; ref. 33). While Bap1 could prevent the accumulation of ubiquitinated SMN in the UPS-inhibited cells, *Bap1*^C91S^ was unable to deubiquitinate SMN (Figure 7E). These results revealed the deubiquitinating function of Bap1 on SMN. Taken together, we concluded that Bap1 directly binds SMN and prevents SMN degradation by posttranslational deubiquitination of SMN.

Since a lysine residue is one of the predominant targets for ubiquitination, we examined which of the lysine residues in SMN is critical for its ubiquitin-dependent degradation by introducing a non-lysine mutation on phylogenetically conserved lysine residues (Supplemental Figure 8A). We found that substitution of the lysine residue at the 186th amino acid to arginine (K186R) substantially prevented ubiquitination of SMN (Figure 7F). This implies that the K186 residue of SMN might be responsible for the ubiquitin-dependent degradation. To investigate the stability of ubiquitination-resistant SMN (SMN^K186R^) in Bap1-null FAPs, we transduced *Rosa-CreER* *Bap1*^fl/fl^ FAPs with lentiviruses delivering either SMN^WT^ or SMN^K186R^, then treated with 4-hydroxytamoxifen (4-OHT) to ablate *Bap1* in these cells (Supplemental Figure 8B). Efficient transductions were confirmed by the in vitro expression of tdTomato, encoded in viral vector (Supplemental Figure 8C), and the ablation of *Bap1* was confirmed by examining genomic DNA recombination, mRNA expression, and protein levels (Supplemental Figure 8, D–F). Although Bap1 was significantly decreased after 4-OHT treatment, mRNA level of *SMN* was unaltered (Supplemental Figure 8G). In the presence of MG132, both SMN^WT^ and SMN^K186R^ remained comparable in both WT and *Bap1*-null FAPs. However, SMN^WT^ showed an apparent decrease in *Bap1*-null FAPs compared with WT FAPs, whereas the amount of SMN^K186R^ remained relatively stable in the absence of MG132 (Figure 7G). These results indicated the predominant degradation of SMN^WT^ without Bap1, consistent with the decreased level of endogenous SMN in *Bap1*^ΔMPC^ FAPs (Figure 7B), and revealed that SMN^K186R^ was resistant to ubiquitination-dependent degradation. In addition, a cycloheximide chase assay revealed that SMN^K186R^ had a prolonged half-life compared with SMN^WT^ in the absence of Bap1 in FAPs (Figure 7H). Collectively, these results indicate that ubiquitin-dependent degradation of SMN requires ubiquitination at K186, and Bap1 prevents the degradation of SMN through its deubiquitination function (Figure 7I).

### Rescue of Bap1^ΔMPC^ mice by SMN^K186R^-expressing FAPs.

**Figure 8:**
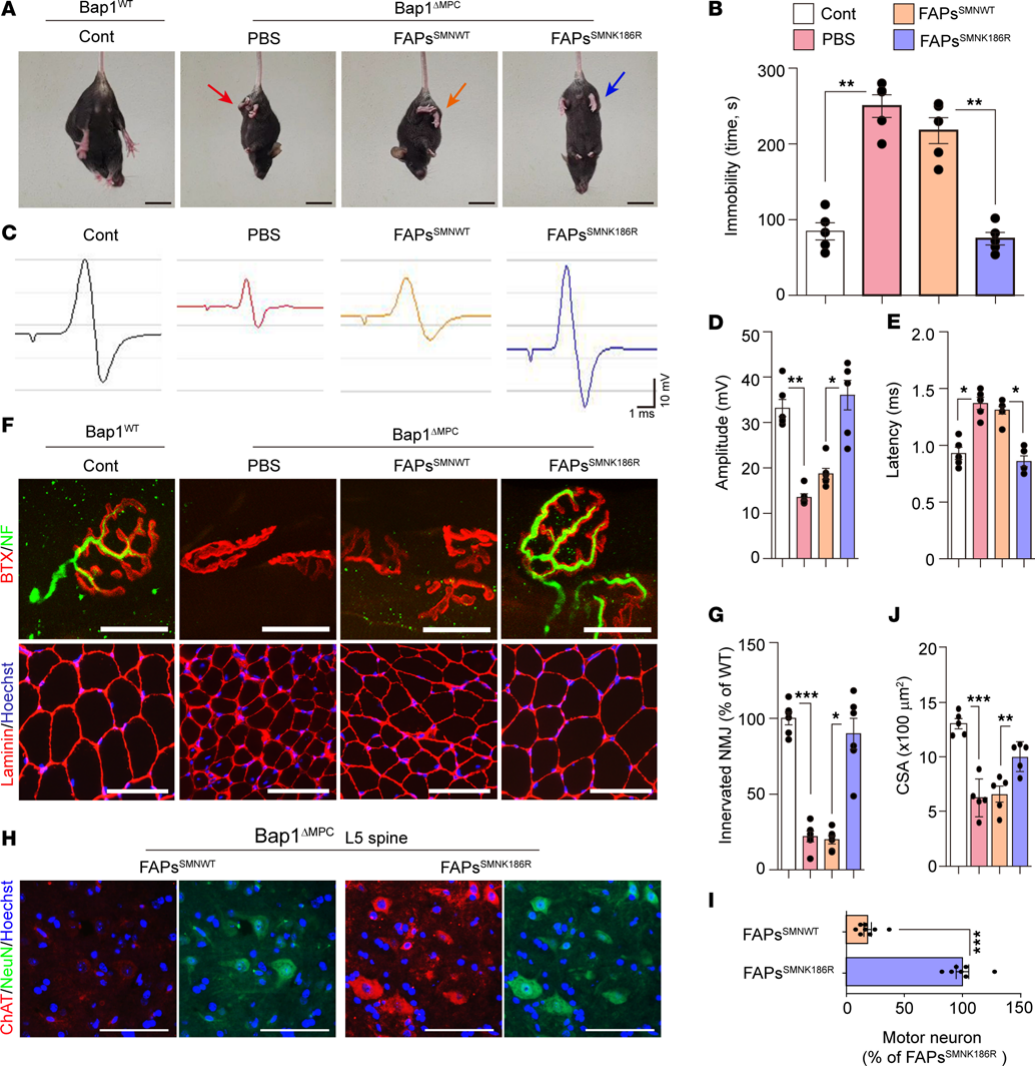
Rescue of *Bap1*^ΔMPC^ mice by SMN^K186R^-expressing FAPs. (**A**) Representative captures of hind limb clasping during tail suspension test. See also Supplemental Video 5. (**B**) Quantification of immobility time during tail suspension test for 5 minutes. (**C**) Representative graph data of CMAP. (**D** and **E**) Quantification of amplitude (**D**) and latency (**E**) of CMAP on GA muscles. (**F**) Representative confocal images for BTX and NF (**F**, top) and IHC staining (**F**, bottom) of TA muscles. (**G**) Quantification of innervated NMJs. (**H**) Representative images of ChAT and NeuN in the ventral horn of L5 spinal cord. (**I**) Quantification of the number of MNs in L5 spinal cord. (**J**) Quantification of CSA in TA muscles. (**B**, **D**, **E**, **G**, **I**, and **J**) *n* = 5 animals for each group; data are mean ± SEM; Tukey’s pairwise comparison test after 1-way ANOVA; *P < 0.05, **P < 0.01, ***P < 0.001. (**A**–**J**) tail suspension tests, CMAP measure, and histological analyses were performed at 8 or 10 weeks after transplantation. Scale bars: 2 cm (**A**), 25 μm (**F**, top), 100 μm (**F**, bottom), 50 μm (**H**).

To investigate whether the dysregulation of SMN deubiquitination in FAPs is directly attributed to the neuromuscular defects in *Bap1*^ΔMPC^ mice, we performed cell transplantation experiments using *Bap1*^ΔMPC^ FAPs expressing either SMN^WT^ or SMN^K186R^. We first examined the recovery of SMN function in *Bap1*^ΔMPC^ FAPs after lentiviral transduction of *SMN*^WT^ or *SMN*^K186R^ (Supplemental Figure 9A). Although the viral transductions induced an excessive mRNA expression of *SMN*, the levels of the snRNAs regulated by SMN were significantly increased only in the *SMN*^K186R^-expressing *Bap1*^ΔMPC^ FAPs (hereafter, FAPs^SMNK186R^), but not in the *SMN*^WT^-expressing *Bap1*^ΔMPC^ FAPs (hereafter, FAPs^SMNWT^) (Supplemental Figure 9, B and C). These results indicated that the degradation-resistant SMN^K186R^, but not SMN^WT^, could function properly in *Bap1*^ΔMPC^ FAPs. Next, to examine whether *SMN*^K186R^ can also restore the in vivo function of *Bap1*^ΔMPC^ FAPs, we transplanted FAPs^SMNWT^ or FAPs^SMNK186R^ into the hind limb muscles of 1.5-week-old *Bap1*^ΔMPC^ mice (Supplemental Figure 9D). Intriguingly, while FAP^SMNK186R^-transplanted *Bap1*^ΔMPC^ mice showed considerable improvements in motor function during tail suspension tests, which became comparable to that of *Bap1*^WT^ mice, FAP^SMNWT^-transplanted *Bap1*^ΔMPC^ mice did not show such improvements (Figure 8, A and B, and Supplemental Video 5). Moreover, decreased amplitude and delayed latency of CMAP in *Bap1*^ΔMPC^ mice were completely restored by FAP^SMNK186R^ but not by FAP^SMNWT^ (Figure 8, C–E). Histological analysis of NMJs also showed rescue effects on AChR cluster morphology and degree of innervation by FAP^SMNK186R^ transplantation only (Figure 8, F and G). Furthermore, motor neuron loss was also prevented only by FAP^SMNK186R^ (Figure 8, H and I). Finally, we observed the increase of CSA only in TA muscles from FAP^SMNK186R^-transplanted *Bap1*^ΔMPC^ mice (Figure 8, F and J). These results revealed that SMN is fundamentally required for the in vivo neuromuscular function of FAPs and that Bap1 maintains a sufficient level of SMN in FAPs by protecting SMN from UPS-dependent degradation.

## Discussion

Understanding the pathogenic mechanisms that cause neurodegeneration in SMA will enable the development of novel therapeutic targets. Here, we showed that Bap1, a deubiquitinating enzyme, is essential for the posttranslational regulation of SMN in FAPs and its disruption leads to the reduction of SMN. Importantly, insufficient levels of SMN in non-neuronal FAPs caused the degeneration of the neuromuscular system and the dying-back loss of motor neurons in spinal cord. Profoundly, however, these degenerations could be completely rescued by the expression of ubiquitination-resistant *SMN*^K186R^ in *Bap1*-null FAPs. Since motor neuron degeneration is the primary manifestation observed in SMA, our finding that the Bap1/SMN pathway is required in non-neuronal FAPs for the maturation and maintenance of the neuromuscular system sheds light on SMA pathogenesis and provides an additional therapeutic strategy to treat SMA.

### FAPs as the non-neuronal peripheral cells for the neuromuscular system.

SMA patients and mouse models show rapid degeneration of motor neurons at disease onset that causes postnatal neuromuscular defects. Since the presynaptic pathologies, such as defective innervation, neurofilament accumulation, synaptic-vesicular defects, and defective ion channels in axonal terminals, are apparent during the progression of the disease (41, 50–53), the insufficient SMN in motor neurons has been considered the main cause of SMA pathogenesis. In line with this, SMN depletion in motor neurons causes motor neuron–intrinsic defects (54, 55). However, genetic studies using loss- and gain-of-function models revealed the marginal requirement of SMN in motor neurons and suggested the involvement of non-neuronal cells in the neuromuscular degeneration. Experiments using systemic delivery of antisense oligonucleotide with the delivery of decoy oligonucleotide into the CNS also revealed that peripheral SMN restorations can completely rescue the degeneration of the neuromuscular system and the loss of motor neurons in SMA mouse models (7, 8, 56). To date, many studies using SMA mouse models implicated several peripheral tissues requiring SMN, such as liver, heart, pancreas, bone, and skeletal muscle, in SMA pathogenesis (57–62). However, it was difficult to directly link these findings to the neurodegeneration of SMA. Through the present work, using genetic and cell transplantation models, we directly showed how FAPs, the non-neuronal peripheral cells in skeletal muscle, not only drive maturation of the neuromuscular system at postnatal growth but also maintain the intact neuromuscular system throughout adulthood. These results indicate that FAPs in skeletal muscle are a fundamental cell population for the neuromuscular system and suggest that its malfunction could be a direct reason for the neurodegeneration in SMA. Thus, the pathogenic effect of abnormal FAPs on SMA is sought to be investigated for a precise understanding of the pathogenic mechanism. In addition, the current human clinical trials have not yet tested the rescue effect of systemic delivery of nusinersen, the drug name of antisense oligonucleotide, for patients with SMA. It can be inferred from the preclinical studies using mouse models that restoration of SMN in both the periphery and the CNS could rescue patients with SMA more dramatically than the current treatment method of intracerebroventricular (ICV) injection, which can deliver drugs into the CNS. Based on these clinical results, we suggest that the restoration of FAPs’ function could have a synergistic rescue effect with the ICV injection of nusinersen. If restoring the function of these cells, such as by transplantation of FAPs in SMA patients, together with the intrathecally delivered nusinersen shows a synergistic rescue effect, our finding will have provided a significant contribution to the development of an improved SMA therapy.

The NMJ has been known as the tripartite synapse composed of presynaptic axon terminals of motor neurons, Schwann cells covering the presynaptic axon terminals, and postsynaptic AChR cluster endplates in myofibers (27, 63–67). Neuromuscular degenerations from NMJ defects are typical for many neuromuscular diseases, including SMA (68, 69). We proposed the FAPs as an additional critical regulator of the neuromuscular system; however, the functional mechanism of this cell population in the regulation of the neuromuscular system still needs to be elucidated. As the FAPs reside between myofibers in the skeletal muscle, they might directly affect the function of NMJ components or be involved in the interactions between NMJ components through direct or paracrine interactions. We suspect SMN to have a regulatory role in the expressions of membrane proteins or secretory factors in FAPs, as SMN is essential for the biogenesis of the spliceosomal small nuclear ribonucleoproteins, which contribute to the posttranscriptional regulation of its targets (70–72). Thus, further studies on revealing the action mechanism of FAPs on the neuromuscular system would be crucial to understanding its establishment and maintenance.

### Physiological roles of FAPs in development and homeostasis.

Skeletal muscle tissues comprise myofibers, muscle stem cells (MuSCs), immune cells, endothelial cells, motor/sensory nerves with Schwann cells, and FAPs. Each cell population plays a unique role in muscle physiology and homeostasis: MuSCs regenerate damaged myofibers upon injury throughout life; immune cells, mostly macrophages, play fundamental roles in the repair and clearance of pathogens and debris from contractile activity or muscle injury; endothelial cells supply nutrients and oxygen across blood vessels; and motor/sensory neurons innervate myofibers to induce contraction and relaxation. However, the roles of FAPs have not been fully elucidated. These cells have been referred to as FAPs, termed according to their differentiation potential in vitro (24). FAPs support muscle regeneration upon muscle injury and sense denervation resulting in muscle atrophy. Meanwhile, they have also been implicated as major contributors to ectopic fat cell formation and fibrosis in aged skeletal muscles (24, 73). Since adipo-fibrotic differentiation of FAPs compromises muscle function (74, 75), it is unlikely that the primary function of FAPs is to differentiate into adipocytes and/or fibroblasts. Based on our data that FAPs play a critical role in the maturation and maintenance of the neuromuscular system, we believe that some of the MPCs should migrate into the muscle tissue during developmental myogenesis and reside there as FAPs for life to maintain muscle integrity (19, 21–24).

In addition, recent studies using single-cell transcriptomics analyses identified 2 subpopulations within skeletal muscle, FAP1 and FAP2, described as Dpp4^–^ FAPs and Dpp4^+^ FAPs in our study, respectively (30, 31, 76). Cluster analysis revealed that Dpp4^–^ FAPs highly express ECM-related genes and that Dpp4^+^ FAPs are enriched for transcripts involved in diverse biological programs. These results suggest the possibility of distinct functions of these 2 subpopulations, but the functional differences have not been investigated. In this study, we identified the role of Dpp4^+^ FAPs in the neuromuscular system, distinct from Dpp4^–^ FAPs, which express ECM-related genes. Our results suggest that while FAP1 may contribute mainly to the fibrosis observed in dystrophic muscles, FAP2 serves as a regulator of the neuromuscular system, ensuring the maturation and maintenance of the NMJ.

Meanwhile, recent studies suggested that FAPs play a role in homeostatic conditions (27, 29). The disruption of FAPs using *Pdgfra-CreER* *Rosa-DTA* models resulted in significant muscle loss and disturbance of NMJs after long-term tracing. However, these results cannot exclude secondary effects due to the activation of the Pdgfra promoter in other cell populations, including pericytes (77–79). Our 4 independent reconstitution experiments with WT FAPs, Dpp4^+^ FAPs, *SMN*^K186R^ expressing *Bap1*^ΔMPC^ FAPs, or *Rosa-CreER* *Bap1*^fl/fl^ FAPs revealed the physiological importance of FAPs in the maturation and maintenance of the neuromuscular system. Most importantly, inactivation of Bap1 in the transplanted *Rosa-CreER* *Bap1*^fl/fl^ FAPs in adults resulted in prompt degeneration of the neuromuscular system, indicating the essential requirement of non-neuronal FAPs in the maintenance of the functional neuromuscular system. Our data suggest that FAPs should be maintained in the skeletal muscle despite the risk of differentiation into adipocytes or fibroblasts in old age.

### Posttranslational regulation of SMN by Bap1.

The UPS regulates the balance in protein homeostasis by mediating a protein degradation process. The role of the UPS in protein homeostasis is fundamental in performing cellular functions. Ubiquitination by the UPS occurs through the coordinated activity of several enzymes, including E1 ubiquitin-activating enzyme, E2 conjugating enzyme, E3 ligase, and DUB. Many different variants of each enzyme have been described: 26 for E1 ubiquitin-activating enzyme, over 105 for E2 conjugating enzyme, ~1003 for E3 ligase, and more than 148 DUBs in eukaryotes (80). Since many neuromuscular diseases show an imbalance in protein homeostasis, researchers have been interested in the function of the UPS in neuromuscular diseases (81). The involvement of the UPS in SMA has also been reported previously: e.g., inhibition of UPS in patient-derived fibroblasts increases intracellular SMN levels, whereas treatment with a ubiquitination inhibitor increases SMN protein levels and slows the progression of disease in severe SMA mouse models (15, 82). Furthermore, such studies revealed the direct regulation of SMN by E3 ligases. Although ubiquitination by ligases is fundamental for the UPS, increasing evidence supports the importance of DUBs as they proofread ubiquitin-protein conjugates and remove unwanted ubiquitinations, contributing to proteostasis. Accordingly, the identification of DUBs regulating SMN homeostasis is required to reveal the mechanisms underlying SMA pathogenesis. Han et al. (83) recently revealed the function of the DUB Usp9x in the regulation of SMN level by showing that this DUB directly interacts with SMN and that knockdown of Usp9x increases SMN levels. Although we believe this was the first study in which a DUB regulating SMN levels was discovered, it was only shown in vitro and was not directly correlated to SMA pathogenesis. In this study, we report that the DUB Bap1, also known as UCHL2, directly interacts with SMN and is crucial for the stabilization of SMN in FAPs by deubiquitination, a prerequisite for the maturation of NMJs and prevention of motor neuron degeneration. To our knowledge, this is a significant in vivo report revealing DUB regulation of the SMN level, which refines our understanding of SMA pathogenesis and provides important insights regarding the treatment of SMA.

## Methods

### Animals.

*Prx1-Cre* (stock 005584), *Rosa-CreER* (stock 008463), *Rosa-YFP* (stock 006148), and *Rosa-tdTomato* (stock 007914) mice were obtained from The Jackson Laboratory. *Bap1*^fl/fl^ mice were generated using the gene trap embryonic stem cell line (HEPD0526_2_G01) made by EUCCOM (the International Knockout Mouse Consortium). In the mutant embryonic stem (ES) cell line, the gene trap cassette was integrated in the fifth intron of the murine *Bap1* locus (Supplemental Figure 1A). The FRT cassette was removed by crossing with flippase-transgenic mouse. Cre-mediated deletion of exons in *Prx1-Cre* *Bap1*^fl/fl^ mice resulted in a frameshift mutation, leading to premature termination by open reading frame. Genotyping primers were designed outside the nondeleted common exon (P1) and were used in combination with primers (P2) within and outside the deleted exon (P3), which could represent genomic deletion (Supplemental Figure 1, A and C). All mouse lines were backcrossed into C57BL/6J background and were housed under specific pathogen–free conditions and handled according to the guidelines of the Institutional Animal Care and Use Committee (IACUC) at Seoul National University. Experiments were carried out with male and female mice, except for the weight analyses and whole transcriptomic analysis, where only male mice were used. No sex-specific differences were observed.

### Behavior test.

All behavior tests were performed by the observers by a protocol blinded to mouse genotypes and descriptions. To evaluate motor functions, previous methods were modified and applied (38, 84, 85). For negative geotaxis assay, mice were placed on a 35° inclined platform with the tail directed up. The latency to turn 180° with the head pointing up the incline was recorded, with cutoff time of 180 seconds. The negative geotaxis score was calculated as fall down the incline or fail to turn within 60 seconds (4 points), turn within 30 seconds (3 points), turn within 20 seconds (2 points), and turn within 10 seconds (1 point). For tail suspension test, mice were suspended by their tail for 3 or 5 minutes. The time that each mouse showed abnormal hind limb clasping or remained immobile was measured. Abnormal hind limb clasping was determined by the proximity of hind-limbs with postural abnormalities. The number of tremor events was quantified on 7-day-old *Bap1*^WT^ and *Bap1*^ΔMPC^ mice hung by their tail for 30 seconds. All behavior tests were conducted between 1500 and 1700 hours to exclude any unwanted circadian effects.

### CMAP measurement.

CMAP of GA muscle was recorded using a previously described protocol with modifications. Briefly, mice were anesthetized using avertin and placed in prone position. Stimulating needle electrodes (A-M Systems) were inserted subcutaneously on both sides of the sciatic notch. The recording electrode (iWorx) was carefully placed subdermally on the GA muscle without puncturing the underlying muscles. A reference electrode and ground electrode were placed near the Achilles tendon and the tail, respectively. The sciatic nerve was stimulated by 1 pulse/s with 0.1 ms duration applying supramaximal stimuli (supramaximal stimulus is about 60 mA) and the following CMAP was recorded. Latency was determined by the delay between the stimulating pulse and the onset point of CMAP response. Amplitude was determined by the absolute difference in maximum positive to maximum negative peak of the responding potential. The CMAP latency and amplitude of an individual animal represent the average of 6 individual measurements of right and left legs.

### Transmission electron microscopy.

Mouse sciatic nerves were fixed with 3% glutaraldehyde in 0.1 M cacodylate buffer (pH 7.2) containing 0.1% CaCl_2_ for 3 hours at room temperature. After washing with 0.1 M cacodylate buffer, they were postfixed with 1% osmium tetraoxide in 0.1 M cacodylate buffer (pH 7.2) containing 0.1% CaCl_2_ for 2 hours at 4°C. Then, nerves were dehydrated with serial ethanol and propylene oxide at 4°C. The samples were embedded in epoxy resin (Embed-812, Electron Microscopy Sciences). Ultrathin sections (70 nm) were prepared with a diamond knife on an ultramicrotome (ULTRACUT UC7, Leica) and mounted on 200 mesh copper grids (EMS). Sections were stained with 2% uranyl acetate for 10 minutes and lead citrate for 3 minutes, then observed using a transmission electron microscope (80 kV, JEM1010, JEOL). The average radius of the area occupied by both myelin sheaths and axons is (D1 + D2)/2, and the average radius of the area occupied by the axons is (d1 + d2)/2. The g-ratio was obtained by (d1 + d2)/2 dividing into (D1 + D2)/2.

### Hind limb interstitial mesenchymal cell isolation.

Isolation of skeletal muscle FAPs, MuSCs, and double-negative cells was performed according to a previously reported protocol (86, 87) with modifications. Limb muscles were dissected and mechanically dissociated in DMEM containing 10% horse serum (Hyclone), collagenase II (800 U/mL; Worthington), and dispase (1.1 U/mL; Thermo Fisher Scientific) at 37°C for 60 minutes. Digested suspensions were subsequently triturated by sterilized syringe with 20G 1/2 needle (BD Biosciences) and washed with DMEM to harvest mononuclear cells. Mononuclear cells were stained with corresponding antibodies. All antibodies used in FACS analysis were listed in Supplemental Table 1. To exclude dead cells, 7-aminoactinomycin D (7-AAD) (MilliporeSigma) was used. Stained cells were analyzed and 7-AAD^–^Lin^–^Vcam^–^Sca1^+^ (FAP), 7-AAD^–^Lin^–^Vcam^+^Sca1^–^ (MuSC), and 7-AAD^–^Lin^–^Vcam^–^Sca1^–^ (double-negative) cells were isolated using FACSAria III cell sorter (BD Biosciences) with 4-way purity precision. Isotype control density plots were used as a reference for positive gating. Fluorescence density plot and population hierarchy of the cells were analyzed using FACSDiva (BD Biosciences) or FlowJo software (BD Biosciences).

### RNA extraction and measurement of mRNA expression.

Total RNA was extracted from skeletal muscles, FAPs, or lentivirus-transduced cells using an RNeasy Micro Kit (QIAGEN) or TRIzol Reagent (Life Technologies) and analyzed by quantitative real-time PCR (qRT-PCR). First-strand complementary DNA was synthesized from 0.1–1 μg of RNA using ReverTra Ace (Toyobo) containing random oligomer according to the manufacturer’s instructions. qRT-PCR (QIAGEN) was performed with SYBR Green technology (SYBR Premix Ex Taq, QIAGEN) using specific primers against indicated genes. Relative mRNA levels were determined using the 2^-ΔΔCt^ method and normalized to *Gapdh* (Figure 7A; Supplemental Figure 1D; Supplemental Figure 8, E and G; and Supplemental Figure 9B), *Chrna1* (Figure 2H), *5.8S rRNA* (Supplemental Figure 7E and 9C), and *b-actin* (Supplemental Figure 5D). Primers are listed in Supplemental Table 1. For RNA-Seq, total RNA was extracted from *Bap1*^WT^ or *Bap1*^ΔMPC^ FAPs using TRIzol Reagent, and TruSeq technology was used to generate Illumina RNA-Seq libraries, according to the manufacturer’s instructions. RNA-Seq libraries were sequenced as paired ends on an Illumina HiSeq 4000 system. DEGs were identified using an FDR cutoff of 1 × 10^−5^. Hierarchical clustering analysis, GO analysis, and GO enrichment analysis were performed using gene expression values from *Bap1*^WT^ versus *Bap1*^ΔMPC^ FAPs. RNA-Seq data sets have been deposited in National Center for Biotechnology Information’s Gene Expression Omnibus (GEO) database under the accession number GSE153826.

### Single-cell RNA sequencing and gene set enrichment analysis.

Freshly isolated FAPs from 3-week-old *Bap1*^WT^ and *Bap1*^ΔMPC^ mice were collected as described above. The scRNA-Seq libraries were generated using 10x Genomics Chromium Next GEM Single Cell 3′ RNA library v3.1 kit, following the manufacturer’s instructions. A total of 5000 cells were targeted for recovery from each genotype of mice. The libraries were sequenced using Illumina’s NovaSeq platform, and the sequenced reads were aligned using CellRanger 3.1.0. software. Using Seurat package 3.2.1 (88) in R version 3.6.1, filtered barcodes and count matrices from CellRanger output were preprocessed for quality control and doublet gating. To exclude the contamination of non-FAPs owing to the limitation in FACS accuracy, cells with high expression levels of the marker genes known to be expressed in muscle-resident cells other than FAPs were removed from further analysis. Dimensionality reduction was performed on merged and normalized data (*Bap1*^WT^+*Bap1*^ΔMPC^) through principal component analysis using the 5000 variable genes identified, and the top 8 principal components were selected for 2-dimensional UMAP embedding and visualization. Unsupervised clustering of the cells was achieved, which were then categorized into Dpp4^+^ or Cxcl14^+^ subpopulations by the expression patterns of marker genes reported previously. DEGs among the subpopulations were selected (fold change > 3, *P* < 0.05) and subjected to gene set enrichment analysis, where the reference list included genes with average normalized expression of more than 0.01 in all cells in the scRNA-Seq data (~12,000 genes). PANTHER 16.0 statistical overrepresentation test was used, and the following parameters were selected for stringent selection of GOs that may have physiological relevance: organism – Mus musculus, annotation data set – GO biological process complete, test type – Fisher’s exact, correction – Bonferroni’s correction for multiple testing. GO terms related to mesenchymal cells, skeletal muscle, or nervous system with the top hierarchy were selected for analysis and display. The scRNA-Seq data are deposited under GEO accession number GSE190426.

### Cell culture, transfection, and plasmids.

Freshly isolated FAPs were cultured at 37°C in alpha-MEM (Hyclone) supplemented with penicillin (100 U/mL), streptomycin (1 mg/mL), and 20% FBS (Hyclone). HEK293T cell line (ATCC) was grown at 37°C in DMEM (Hyclone) supplemented with antibiotics and 10% FBS. The cell line was tested for mycoplasma contamination. All transfections were performed using polyethyleneimine (PEI; Polysciences) and Metafectene reagent (Biontex) following the manufacturer’s instructions. Full-length and point-mutated constructs of SMN were subcloned into the pcDNA4-HisMax vector. N-terminal flag-tagged full-length, deleted, and point-mutated construct of mouse Bap1 and human BAP1 were subcloned into the pcDNA4 or pcDNA3.1 vector (Addgene). HA-tagged Ub was subcloned into pcDNA3 vector.

### Lentivirus packaging and transduction.

With psPAX2 (4.5 μg) and pMD2.G (1.5 μg), lentiviral pLVX-SMN^WT^-IRES-tdTomato or pLVX-SMN^K186R^-IRES-tdTomato (6 μg) vectors were cotransfected into HEK293T cells using PEI. psPAX2, pMD2.G, and lentiviral pLVX-IRES-tdTomato were purchased from Addgene. pLVX-SMN^WT^-tdTomato and pLVX- or SMN^K186R^-tdTomato vectors were cloned using pLVX-IRES-tdTomato as a backbone DNA. The cultured supernatants containing virus particles were harvested 48 hours posttransfection, filtered through 0.45 μm filters, and concentrated using a Lenti-X concentrator (Takara). For virus titration, quantitative PCR (qPCR) analysis was performed. For standard curve, transgene expressing plasmid was digested with restriction enzymes and run on a 2% agarose gel at 200 V for 20 minutes. A single fragment of defined size was purified using PCR purification kit (Macherey-Nagel) and measured for the DNA concentration. DNA was finally diluted to 1 × 10^9^ vector genomes (vg)/μL. Standard plasmid was 10-fold serially diluted from 1 × 10^7^ vg/μL to 1 × 10^1^ vg/μL in triplicates. For serially diluted template DNA, 2 μL of the concentrated lentiviral stock was mixed with 198 μL of DNaseI buffer containing 2 μL of DNaseI in PCR tubes and incubated for 30 minutes at 37°C. After inactivation of DNaseI, the solution was 10-fold serially diluted from 1 × 10^–3^ vg/μL to 1 × 10^–5^ vg/μL in triplicates. qPCR analysis of diluted DNA was conducted to calculate the concentration of lentivirus. For virus transduction, freshly isolated FAPs and cultured *Bap1*^WT^ or *Bap1*^ΔMPC^ FAPs were centrifuged at 500*g* for 5 minutes and the supernatant was aspirated. The cell pellet was resuspended with fresh media containing lentiviral stock with 4 μg/mL polybrene (Santa Cruz Biotechnology) and then incubated in an appropriate culture dish for 16 hours at 37°C. After incubation, viral media were replaced by alpha-MEM supplemented with penicillin (100 U/mL), streptomycin (1 mg/mL), and 20% FBS. The final 5.0 × 10^8^ vg/mL titer lentiviral particles were used.

### FAP transplantation.

FAPs were purified by FACS from limb muscles of indicated mice. A total of 1 × 10^5^ FAPs were resuspended in 1× PBS and immediately transplanted into both TA and GA muscles of *Bap1*^ΔMPC^ mice, littermates of donor mice. Control mice (*Bap1*^WT^ or *Bap1*^ΔMPC^ mice) received the same volume of PBS.

### Immunoblot and immunoprecipitation.

Freshly isolated FAPs and lentivirus-transduced cells were homogenized in RIPA buffer (50 mM Tris-HCl at pH 7.5, 0.5% SDS, 20 μg/mL aprotinin, 20 μg/mL leupeptin, 10 μg/mL phenylmethylsulfonyl fluoride, 1 mM sodium orthovanadate, 10 mM sodium pyrophosphate, 10 mM sodium fluoride, and 1 mM dithiothreitol). Cell lysates were centrifuged at 15,928*g* for 15 minutes. Supernatants were collected and subjected to immunoblot. Bradford’s reagent (Bio-Rad Laboratories) was used for estimating total protein concentrations. Normalized total proteins were analyzed by electrophoresis in 8% to 10% polyacrylamide gels and transferred to PVDF membranes (MilliporeSigma). Membranes were blocked in 5% skim milk (BD Biosciences) in TBS with 0.1% Tween-20 and incubated with primary antibodies overnight at 4°C. After incubation with the corresponding HRP-conjugated secondary antibodies, the membranes were developed using Fusion solo chemiluminescence imaging system (Vilber). Gapdh, α-tubulin, or β-actin was used as a loading control. Antibodies used in this study are listed in Supplemental Table 1. Primary and secondary antibodies were diluted 1:1000 and 1:10,000 with PBS containing 0.1% Tween-20 and 3% BSA, respectively. For the reciprocal immunoprecipitation assay, freshly isolated FAPs and expressing vector-transfected HEK293T cells were harvested and lysed in IP buffer (150 mM NaCl, 50 mM Tris, 0.5% Nonidet P-40) supplemented with 1× protease inhibitors (Thermo Fisher Scientific) and 1 μg/mL pepstatin. Protein lysates were precleared with protein A/G PLUS-Agarose beads (Santa Cruz Biotechnology) for 1 hour at 4°C. Precleared lysates were incubated with primary antibodies overnight at 4°C. After centrifugation at 800*g* for 2 minutes at 4°C, precipitated proteins were washed with IP buffer, eluted in 2× Laemmli sample buffer followed by heating at 100°C for 10 minutes, and subjected to immunoblot.

### Ubiquitination assay.

HisMax-SMN and Flag-Bap1 plasmids were transfected in HEK293T cells with or without HA-tagged Ubiquitin plasmids. After incubation for 24 hours, cells were treated with MG132 (4 μg/mL) for 4 hours and lysed by boiling for 10 minutes in 150 mM Tris-HCl (pH 8), 5% SDS, and 30% glycerol. Cell lysates were diluted 20-fold with buffer A (20 mM Tris-HCl at pH 8.0, 150 mM NaCl, 1% Triton X-100, 1× protease inhibitor cocktail; Thermo Fisher Scientific) containing 10 mM imidazole and 2 mM *N*-ethylmaleimide. After incubating them with Ni_2_^+^–NTA–agarose (QIAGEN) for 2 hours at 4°C, beads were washed 3 times with buffer A containing 20 mM imidazole, then boiled in SDS sampling buffer. Supernatants were subjected to immunoblot analysis.

### Histochemistry and immunohistochemistry.

GA and TA muscles were embedded in the O.C.T. Compound (Sakura Finetek), snap-frozen in liquid nitrogen, and stored at –80°C prior to sectioning. Embedded muscles were sliced into 7 μm sections using a cryostat (Leica). Basic muscle morphology and centrally nucleated myofibers were assessed with H&E staining according to basic protocol. For staining of muscle sections, randomly selected slides at the same sectional position between groups were fixed in 4% paraformaldehyde (PFA). Fixed slides were blocked for 1 hour at room temperature with blocking buffer (5% goat and horse serum in PBS) and incubated overnight with rat anti-laminin (Abcam, ab11576, 1:2000 dilution) in blocking buffer at 4°C. After incubation, slides were washed with PBS 3 times and were incubated for 1 hour with 1:400 dilution of Alexa Fluor 488–conjugated anti–rat IgG and 1:5000 dilution of Hoechst 33342 (Thermo Fisher Scientific). For spinal cords, L5 spinal cords were isolated and fixed in 4% PFA overnight at 4°C and were incubated in 30% sucrose solution following 15% sucrose solution until the spinal cords sinked. Processed spinal cords were embedded in O.C.T. Compound, snap-frozen in liquid nitrogen, and stored at –80°C until sectioning. Embedded spinal cords were sectioned, 7 μm thick, using a cryostat. For staining, slides of spinal cords were washed with PBS and permeabilized with cold methanol for 30 minutes in 4°C. Samples were put into a staining dish containing preheated (105°C) sodium citrate buffer (10 mM sodium citrate, 0.05% Tween 20, pH 6.0) for 10 minutes and washed 2 times with PBS-0.5% Triton X-100 (PBS-T) and 1 time with PBS for 10 minutes each. Afterward, slides were incubated in mouse IgG blocking reagent for 1 hour at room temperature to block endogenous mouse immunoglobulin and washed 3 times with PBS for 10 minutes each. Sections were then blocked with blocking solution (5% donkey serum; 2% BSA, PBS-T) for 1 hour. Slides were incubated overnight at 4°C with the primary antibody for NeuN and ChAT. Then, slides were washed and incubated with secondary antibody (Alexa Fluor 488 goat anti–mouse IgG, 1:400, Alexa Fluor 594 donkey anti–goat IgG, 1:400) at room temperature for 1 hour. Nuclei were counterstained with Hoechst 33342 (Thermo Fisher Scientific, 1:5000). Slides were mounted with VECTASHIELD (Vector Laboratories) and covered by coverslips. For staining of NMJs, TA and GA were dissected, fixed overnight in 4% PFA at 4°C, and washed 3 times with PBS. Muscle fiber bundles were quenched by 0.1 mM glycine in PBS and teased out and blocked overnight at 4 °C. Muscle bundles were incubated with rabbit anti-neurofilament antibodies in PBS-T overnight at 4°C. After 3 washes with PBS-T, muscles were stained with Alexa Fluor 488–conjugated anti–rabbit IgG (1:400), rhodamine-conjugated BTX (1:500), and Hoechst (1:5000). Finally, muscle fiber bundles were washed 3 times with PBS-T and mounted in VECTASHIELD.

### Optical systems.

Immunofluorescence images were acquired using a Zeiss Observer Z1 fluorescence microscope equipped with a SPOT Flex camera or a Zeiss confocal system LSM710 or Leica SP8. For myofiber diameter measurements, Leopard morphometric software (INS Industry), SPOT software (version 5.1, Diagnostic Instruments), and Photoshop CS6 (Adobe) were used. Images were acquired from 15 distinct cross-sectional fields of each muscle and L5 spinal cord sections. The averages of CSA and number of ChAT^+^ motor neurons in groups were quantified and statistically analyzed. For sciatic nerve g-ratio, inner axonal diameter and total outer fiber diameter were measured by FIJI image analysis software. To quantify innervated NMJs, a minimum of 150 NMJs from each muscle were captured by Leica SP8 confocal laser scanning microscopy. LasX software was used to obtain maximal projections and merged images of the red and green channels. Innervation status was evaluated based on the alignment of BTX (red channel) and neurofilament (green channel). NMJs with >70%~80% of overlapping area of BTX and NF signals were classified as innervated NMJs. The percentages of innervated NMJs were normalized by the number of innervated NMJs in controls.

### Statistics.

All statistical analyses were performed using GraphPad Prism 5 (GraphPad Software). Continuous variables were tested for normal distribution by using the Kolmogorov-Smirnoff test. For comparison of significant differences in multiple groups, statistical analysis was performed by 1-way ANOVA followed by Bonferroni’s or Tukey’s pairwise comparison post hoc test for normally distributed data, or Kruskal-Wallis test with Mann-Whitney *U* post hoc test, as appropriate. For comparison of 2 groups of normally distributed data, Student’s *t* test assuming a 2-tailed distribution were used. For non-normally distributed data, Mann-Whitney *U* test was used. For comparison of 2 groups at multiple time points, multiple unpaired *t* test was used. Unless otherwise noted, all error bars represent mean ± SEM. *P* value of less than 0.05 was considered statistically significant at the 95% confidence level. The number of biological replicates, raw values, and statistical analysis for each experiment were indicated in the figure legends. Independent experiments were performed at least in triplicates.

### Study approval.

The care and treatment of animals were approved by the IACUC of Seoul National University.

## Author contributions

JHK designed this study, conducted the experiments, and wrote the manuscript. JSK and KY conducted the experiments and wrote the manuscript. JJ, IP, JHP, JR, SJ, YWJ, SHH, MS, and SM conducted the experiments. SJU and RHS provided critical materials and helpful comments for this study. YYK designed the research and wrote the manuscript. All authors read and approved the final manuscript.

## Supplementary Material

Supplemental data

Supplemental video 1

Supplemental video 2

Supplemental video 3

Supplemental video 4

Supplemental video 5

## Version 1 05/23/2022

Electronic publication
