# Supplementary material for: Bap1/SMN axis in Dpp4+ skeletal muscle mesenchymal cells regulates the neuromuscular system
Source: JCI Insight. 2022 May 23;7(10):e158380. doi: 10.1172/jci.insight.158380 (PMC9220848; doi:10.1172/jci.insight.158380)
Supplement: Supplemental data [file jciinsight-7-158380-s022.pdf]

Supplemental figures

Bap1-SMN axis in Dpp4^{+}-skeletal muscle mesenchymal cells regulates the neuromuscular system

Ji-Hoon Kim, Jong-Seol Kang, Kyusang Yoo, Jinguk Jeong, Inkuk Park, Jong Ho Park, Joonwoo Rhee, Shin Jeon, Youngwoo Jo, Sang-Hyeon Hann, Minji Seo, Seungtae Moon, Soo-Jong Um, Rho Hyun Seong, and Young-Yun Kong

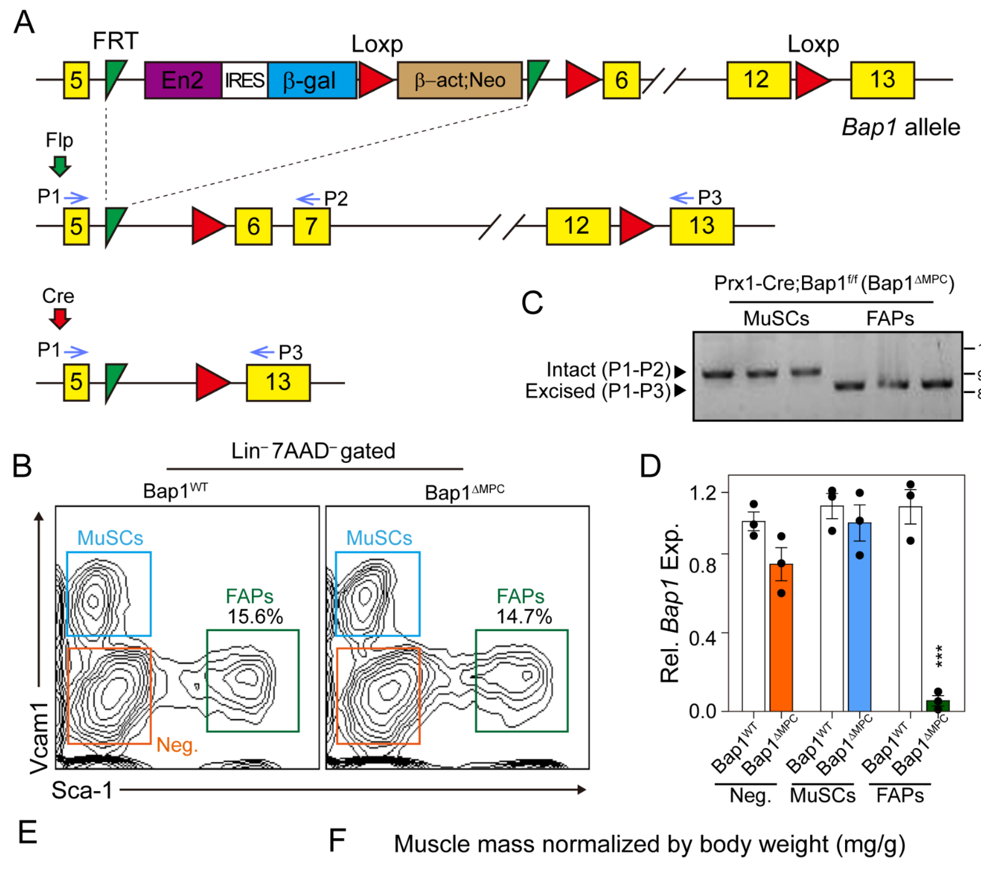

Age (W)	Group	TA	GA	Q	Tri
1W	Bap1^{WT}	0.78±0.07	1.5±0.15	1.41±0.26	1.59±0.22
	Bap1$^{\Delta MPC}$	0.70±0.04	1.37±0.28	1.30±10.37	1.38±0.31
2W	Bap1^{WT}	2.75±0.38	5.04±0.62	4.57±0.65	3.06±0.46
	Bap1$^{\Delta MPC}$	2.33±0.74	3.53±0.39*	3.17±0.59*	2.38±0.30
3W	Bap1^{WT}	4.34±0.99	7.66±1.92	7.08±1.64	3.78±0.86
	Bap1$^{\Delta MPC}$	2.32±0.28*	4.67±0.87*	3.47±0.44*	3.14±0.37
4W	Bap1^{WT}	3.57±0.14	13.45±3.71	7.61±0.62	3.96±0.16
	Bap1$^{\Delta MPC}$	1.11±0.13*	4.12±0.08*	3.84±0.33*	2.17±0.19*
8W	Bap1^{WT}	2.28±0.09	7.34±0.16	6.29±0.46	3.74±0.37
	Bap1$^{\Delta MPC}$	0.48±0.28*	2.31±0.42*	3.60±0.17*	1.93±0.10*
12W	Bap1^{WT}	2.41±0.31	6.91±0.55	6.31±0.76	4.02±0.80
	Bap1$^{\Delta MPC}$	0.56±0.11*	2.28±0.36*	3.16±0.19*	1.24±0.62*

Supplemental Figure 1. Muscle loss by deletion of *Bap1* in somatic lateral plate-derived cells during postnatal growth. (**A**) A scheme for the generation of Bap1 floxed (*Bap1*$^{f/f}$) mice. (**B**) Flow cytometric plots of the lineage-negative (CD31/CD45-negative) and live (7AAD-negative)-gated cells in hindlimb muscles from 1.5-week-old *Bap1*^{WT} or *Bap1*$^{\Delta MPC}$ mice. Note that freshly isolated Lin^{-}Vcam^{-}Sca1^{+} and Lin^{-}Vcam^{+}Sca1^{-} cells were regarded as interstitial mesenchymal cells (FAPs) and muscle stem cells (MuSCs), respectively. Negative cells indicate the Lin^{-}Vcam1^{-}Sca1^{-} population. (**C**) Bap1 deletion in genomic DNA in FAPs from *Bap1*$^{\Delta MPC}$ hind-limb muscles. (**D**) Relative *Bap1* expression in negative cells (orange rectangular), MuSCs (sky blue) and FAPs (green) sorted from 1.5-week-old *Bap1*^{WT} or *Bap1*$^{\Delta MPC}$ hindlimb muscles. n=3 animals for each group; data are mean±s.e.m.; Mann–Whitney U test; ***p<0.001. (**E**) Growth curve of *Bap1*^{WT} and *Bap1*$^{\Delta MPC}$ mice. n=4 animals per group; mean±s.e.m.; Multiple unpaired t-test; *p<0.05, **p<0.01, n.s, not significant. (**F**) Mean muscle mass normalized by body weight (g/mg) of TA, GA, Q, and Tri muscles from *Bap1*^{WT} or *Bap1*$^{\Delta MPC}$ mice at the indicated ages. n=4 animals for each group; data are mean±s.e.m.; Mann–Whitney U test; *p<0.05.

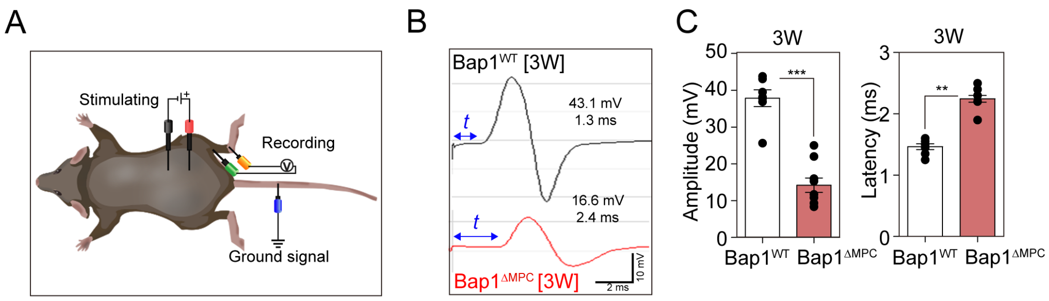

Supplemental Figure 2. Reduced CSA and abnormal CMAP in Bap1$^{\Delta MPC}$ mice. (**A**) A scheme of the CMAP measurement on GA muscles. (**B** and **C**) Representative graph data (**B**) and quantifications of amplitude and latency (**C**) of CMAP on 3-week-old GA muscles of *Bap1*^{WT} or *Bap1*$^{\Delta MPC}$ mice. n=5 animals for each group; data are mean±s.e.m.; Mann–Whitney U test; ***p*<0.01, ****p*<0.001.

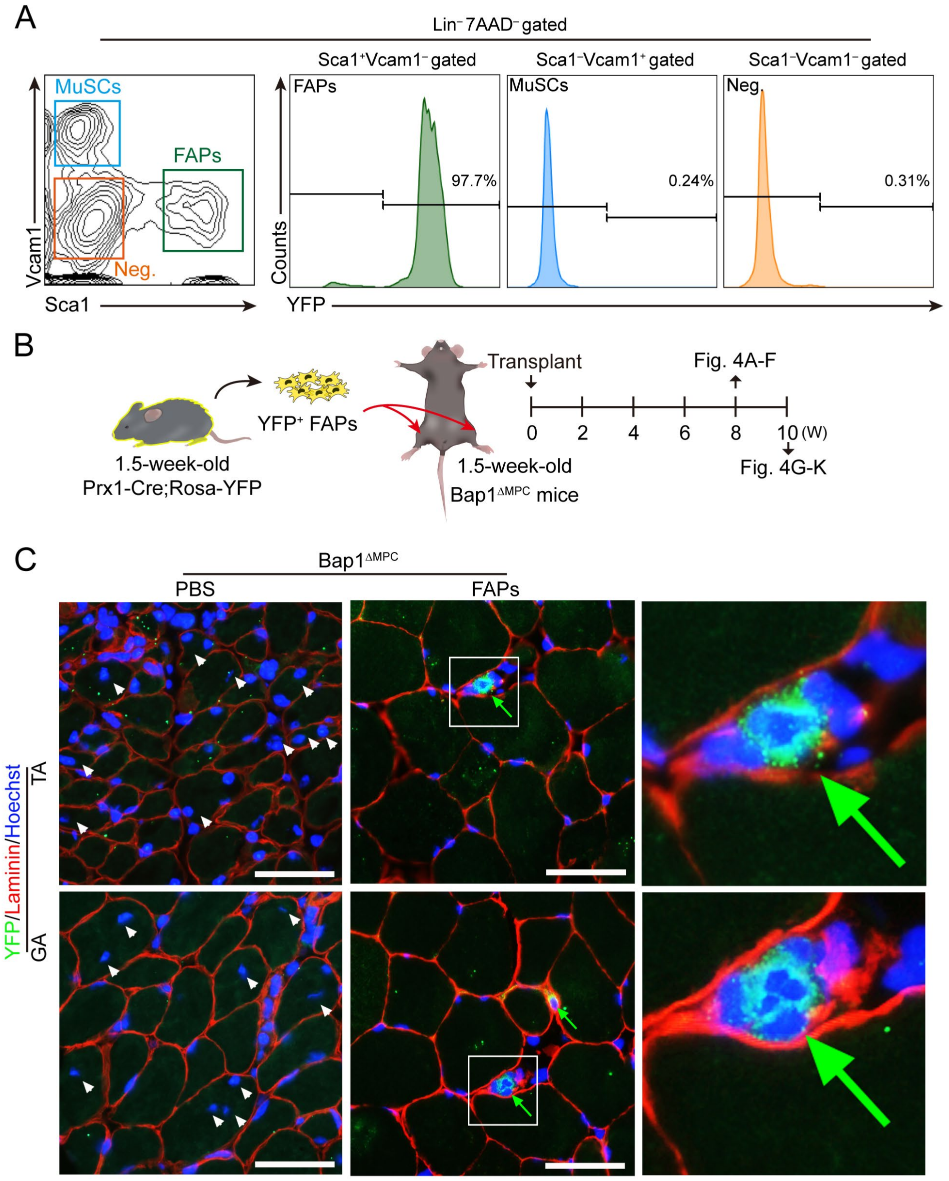

Supplemental Figure 3. Restoration of the neurodegenerative phenotypes on Bap1$^{\Delta MPC}$ mice by wild-type FAPs transplantation. (**A**) Flow cytometric plots of the lineage negative (CD31/CD45-negative) and live (7AAD-negative)- gated cells from hindlimb muscles of 1.5-week-old Prx1-Cre; Rosa-YFP transgenic mice. Note that YFP expression indicates successful recombination by the Cre recombinase in FAPs, indicating that among the major cellular components in the skeletal muscle, only FAPs are derived from the somatic lateral plate mesoderm. (**B**) Experimental scheme for wild-type YFP+ FAPs transplantation. YFP+ FAPs isolated from 1.5-week-old Prx1-Cre; Rosa-YFP mice were transplanted into 1.5-week-old *Bap1*$^{\Delta MPC}$ mice and histological analyses were performed at the indicated weeks after transplantation. (**C**) Representative images of 11.5-week-old *Bap1*$^{\Delta MPC}$ TA muscles transplanted with PBS or FAPs. Arrow YFP+ FAPs. Right panels are magnified images of the indicated rectangular regions in the middle panels. Scale bars; 50 μm.

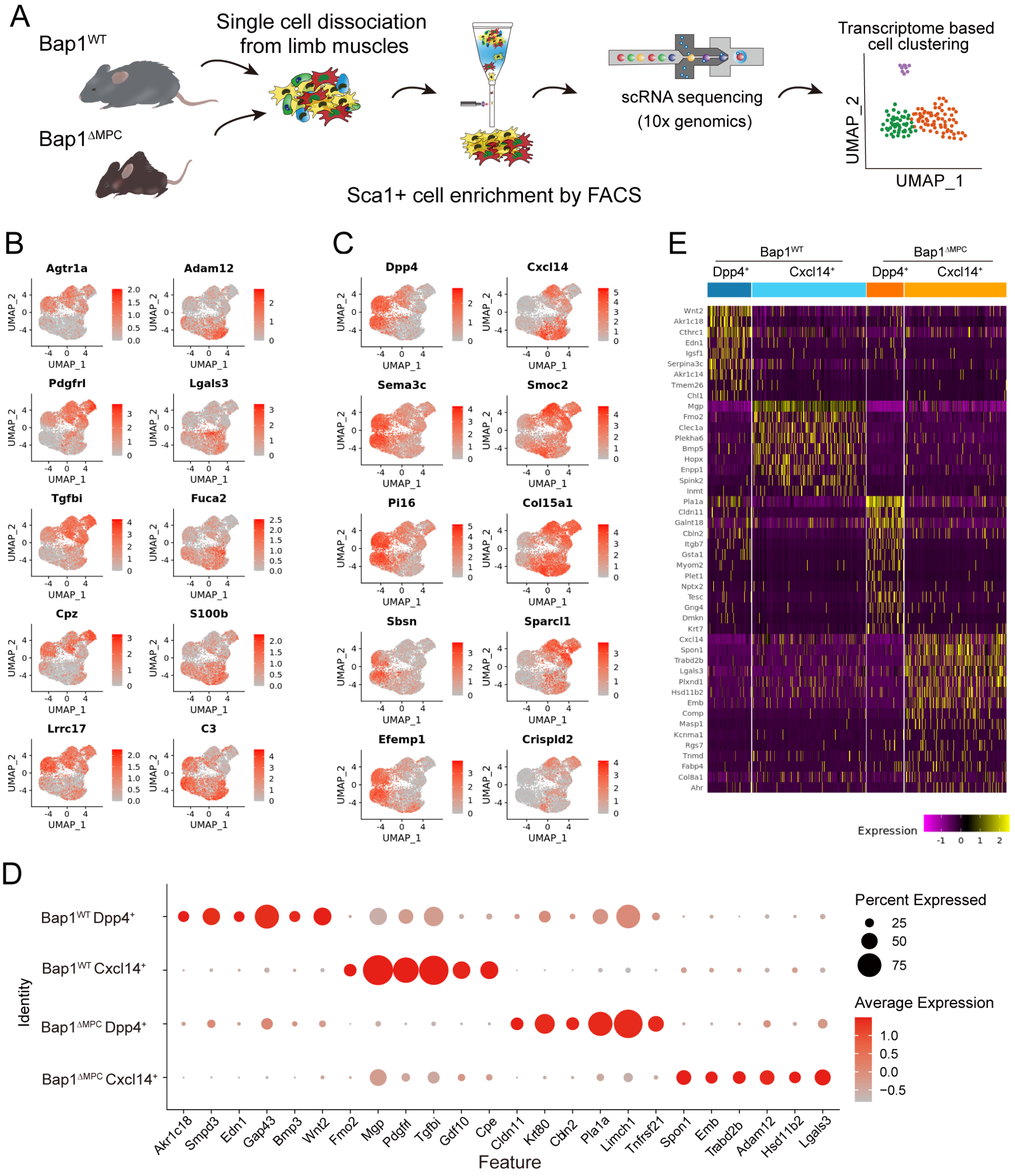

Supplemental Figure 4. Single-cell RNA sequencing analysis on Bap1^{WT} vs Bap1$^{\Delta MPC}$ FAPs. (A) Experimental scheme of scRNA-seq workflow. (**B**) UMAP plots showing the expression levels of top 10 DEGs in Bap1^{WT} vs Bap1$^{\Delta MPC}$ FAPs as a whole. Five genes on the left are upregulated in Bap1^{WT} FAPs, and five on the right in Bap1$^{\Delta MPC}$ FAPs. (**C**) UMAP plots showing the expression levels of 10 marker genes previously reported to separate the Dpp4^{+} subpopulation from the Cxcl14^{+} subpopulation. Five marker genes exclusively expressed in Dpp4^{+} FAPs, including Dpp4 itself, is shown on the left, and five marker genes exclusively expressed in Cxcl14^{+} FAPs, including Cxcl14 itself, is shown on the right. **(D)** Dotplot showing representative genes uniquely expressed in each cluster. (**E**) Top marker genes uniquely expressed in each cluster with a FC > 3 are displayed in heatmap.

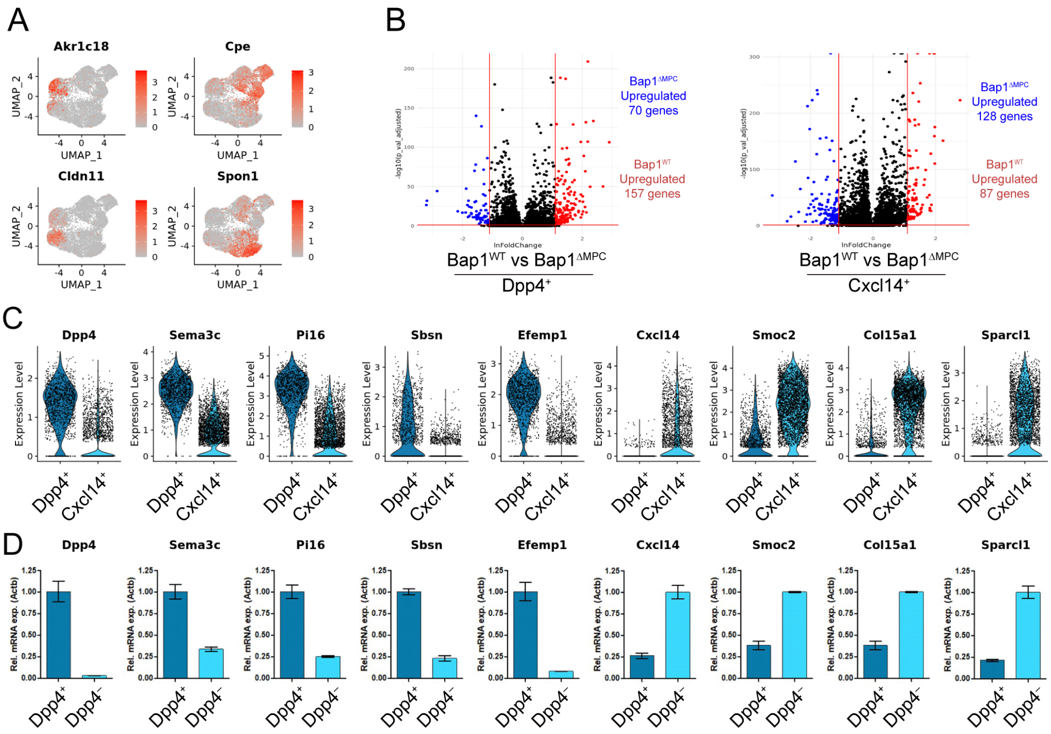

Supplemental Figure 5. Transcriptomic changes of genes related to neurological functions in Dpp4^{+}-*Bap1*$^{\Delta MPC}$ FAPs. (**A**) Example of UMAP plots depicting expression levels of uniquely expressed genes in each cluster. (**B**) Volcano plots highlighting the DEGs selected for downstream GO statistical overrepresentation test. DEGs from comparing Dpp4^{+}-Bap1^{WT} FAPs vs Dpp4^{+}-*Bap1*$^{\Delta MPC}$ FAPs (left) and DEGs from comparing Cxcl14^{+}-Bap1^{WT} FAPs vs Cxcl14^{+}-*Bap1*$^{\Delta MPC}$ FAPs (right) is displayed. (**C**) Violin plots showing expression levels of selected marker genes that separate Dpp4^{+} FAPs from Cxcl14^{+} FAPs. (**D**) Expression of selected marker genes in FACS-sorted Dpp4^{+} vs Dpp4^{-} FAPs from tdTomato reporter mice by RT-qPCR.

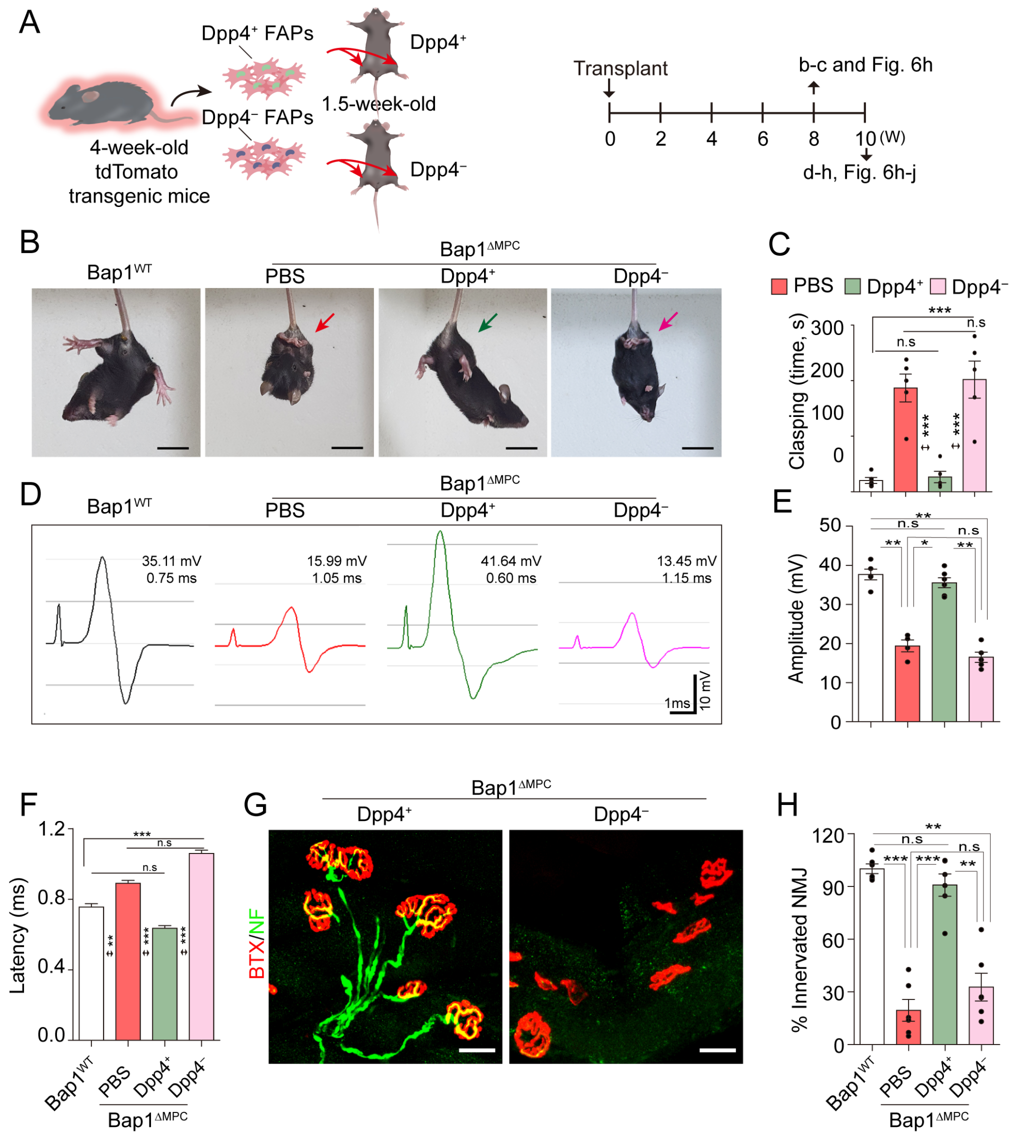

Supplemental Figure 6. Restoration of the neurodegenerative phenotypes in Bap1$^{\Delta MPC}$ mice by Dpp4$^+$ FAPs transplantation. (**A**) Experimental scheme for Dpp4$^+$/Dpp4$^-$ FAPs transplantation. Dpp4$^+$ or Dpp4$^-$ FAPs from 4-week-old tdTomato transgenic mice were transplanted into 1.5-week-old *Bap1*$^{\Delta MPC}$ mice (Dpp4$^+$ or Dpp4$^-$) and histological analyses were performed at the indicated weeks after transplantation. (**B**) Representative captures of hindlimb clasping during tail-suspension test. Arrow indicates the hindlimb clasping. Note that clasping phenotype in *Bap1*$^{\Delta MPC}$ mice (red) was alleviated by Dpp4$^+$ FAPs-transplantation (green), but not by Dpp4$^-$ FAPs-transplantation (pink). See also Supplemental Video 4. (**C**) Quantification of hindlimb clasping time during tail-suspension test. n=5 animals for each group; data are mean±s.e.m; Tukey's pairwise comparison test after one-way ANOVA; ****p*<0.001. (**D**–**F**) Representative CMAP amplitude and latency recordings (**D**) and quantification of amplitude (**E**) and latency (**F**) measured in GA muscles. n=4 animals for each group; data are mean ± s.e.m; Tukey's pairwise comparison test after one-way ANOVA; **p*<0.05, ***p*<0.01, ****p*<0.001; n.s, not significant. (**G**) Representative confocal images of BTX and NF in TA muscles. Scales, 25 μm. (**H**) Quantification of innervated NMJs in TA muscle. *n*=5 animals for each group; data are mean±s.e.m; Tukey's

pairwise comparison test after one-way ANOVA; *p<0.05, **p<0.01. Scale bars; 2cm (**B**), 100 μm (**G**)

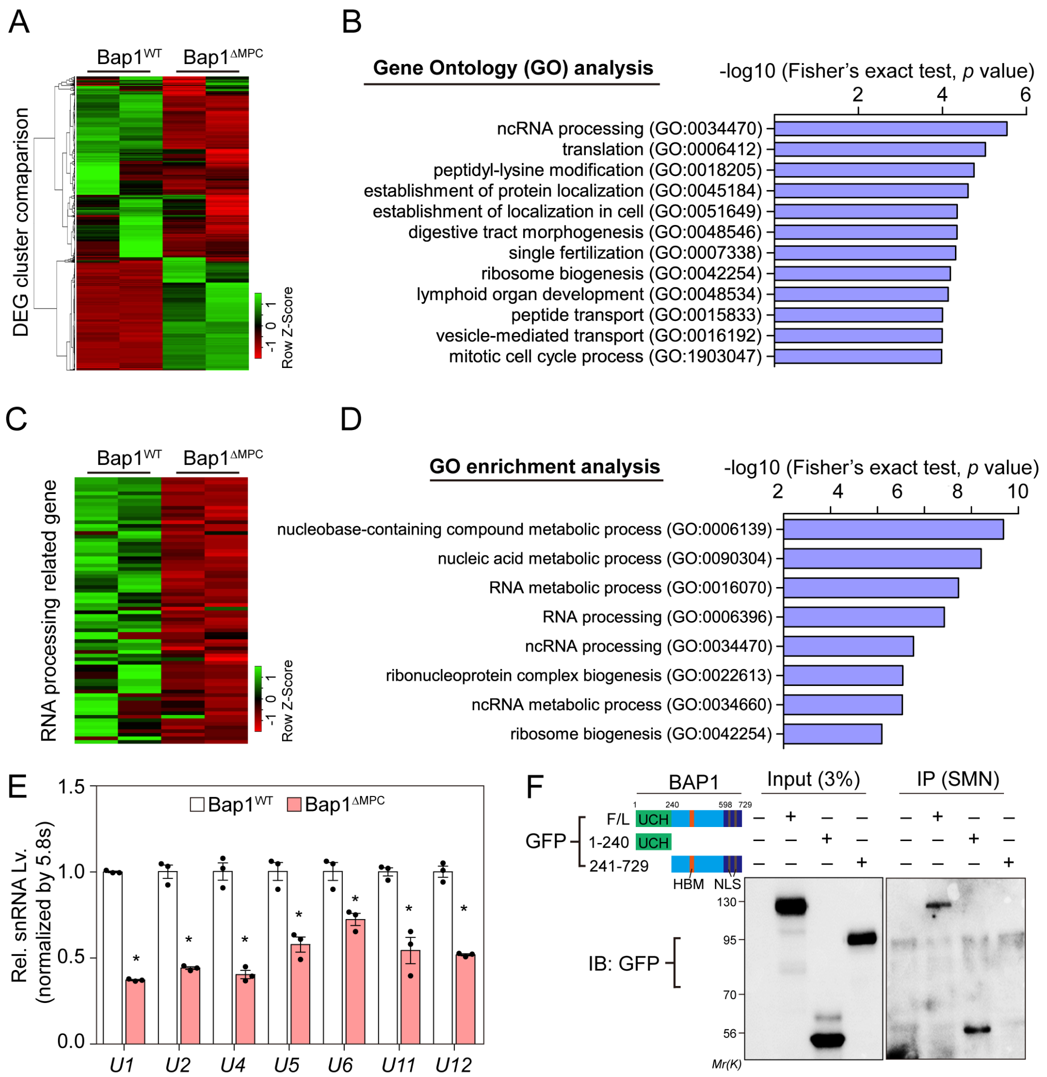

Supplemental Figure 7. A potential association between Bap1 and SMN. (**A**–**D**) Freshly isolated FAPs were analyzed by RNA sequencing. Heat map of differentially expressed genes by the one-way hierarchical clustering (Euclidean Method, Complete Linkage) (**A**). GO analysis (**B**), Heat map (**C**), and GO enrichment analysis (**D**) for significantly down-regulated genes relating to RNA processing. Enrichment analysis was performed on genes with significant changes (fc > 2, down in Bap1$^{\Delta MPC}$ FAPs). Z-score was calculated across the rows. (**E**) Relative snRNA expression in FAPs isolated from 1.5-week-old *Bap1*^{WT} and *Bap1*$^{\Delta MPC}$ hind-limb muscles. n=4 animals for each group; data are mean±s.e.m.; Mann–Whitney U test; *$p<0.05$. (**F**) Semi-endogenous immunoprecipitation of SMN and BAP1 truncated forms. GFP-BAP1 full-length (GFP-BAP1$^{F/L}$), GFP-BAP1$^{1\text{-}240}$ and GFP-BAP1$^{241\text{-}729}$ expression vectors were transiently transfected in HEK293T cells. Twenty-four hours after transfection, cell lysates were immunoprecipitated with anti-SMN antibody and subjected to immunoblot analyses with an anti-GFP antibody.

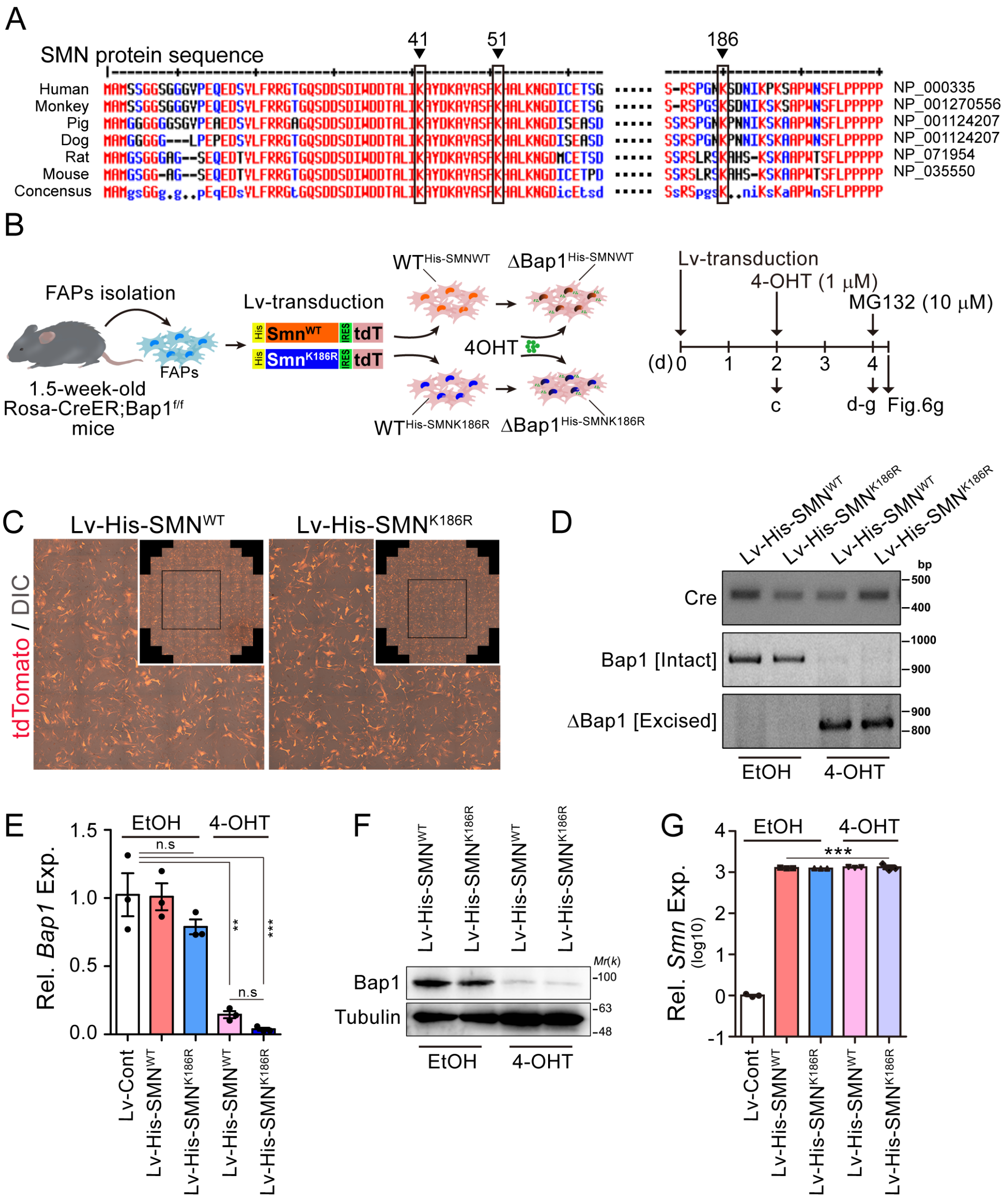

Supplemental Figure 8. Establishment of Bap1 null FAPs over-expressing SMN^{WT} or SMN^{K186R}. (**A**) Sequence alignments of SMN orthologs from multiple species. The lysine residues, which are potential ubiquitination sites of SMN1 protein, are well conserved between human, monkey, pig, dog, rat and mouse. (**B**–**G**) FAPs purified from 1.5-week-old Rosa-CreER; *Bap1*$^{f/f}$ hind-limb muscles were transduced with lentiviral vectors containing His-tagged SMN^{WT}-IRES-tdTomato (Lv-His-SMN^{WT}) and His-tagged SMN^{K186R}-IRES-tdTomato (Lv-His-SMN^{K186R}). To delete *Bap1* gene, lentivirus-transduced FAPs were treated with 1 μM of 4-OHT for 48 hours. An experimental scheme (**B**). Fluorescent images for tdTomato expression (**C**). PCR analysis of the *Bap1* deletion on genomic DNA obtained from 4-OHT-treated Lv-His-SMN^{WT} and Lv-His-SMN^{K186R} FAPs (**D**). Note that the excised band is only detected in 4-OHT-treated FAPs. Relative Bap1 mRNA (**E**) and protein (**F**) expression in the FAPs in the absence or presence of 4-OHT treatment. Relative SMN mRNA expression in the FAPs (**G**). Note that transduced SMN lentiviral constructs were well expressed even after 4-OHT treatment. n=4 animals for each group; data are mean±s.e.m;

Tukey's pairwise comparison test after one-way ANOVA; *$p<0.05$, **$p<0.01$, ***$p<0.001$; n.s, not significant.

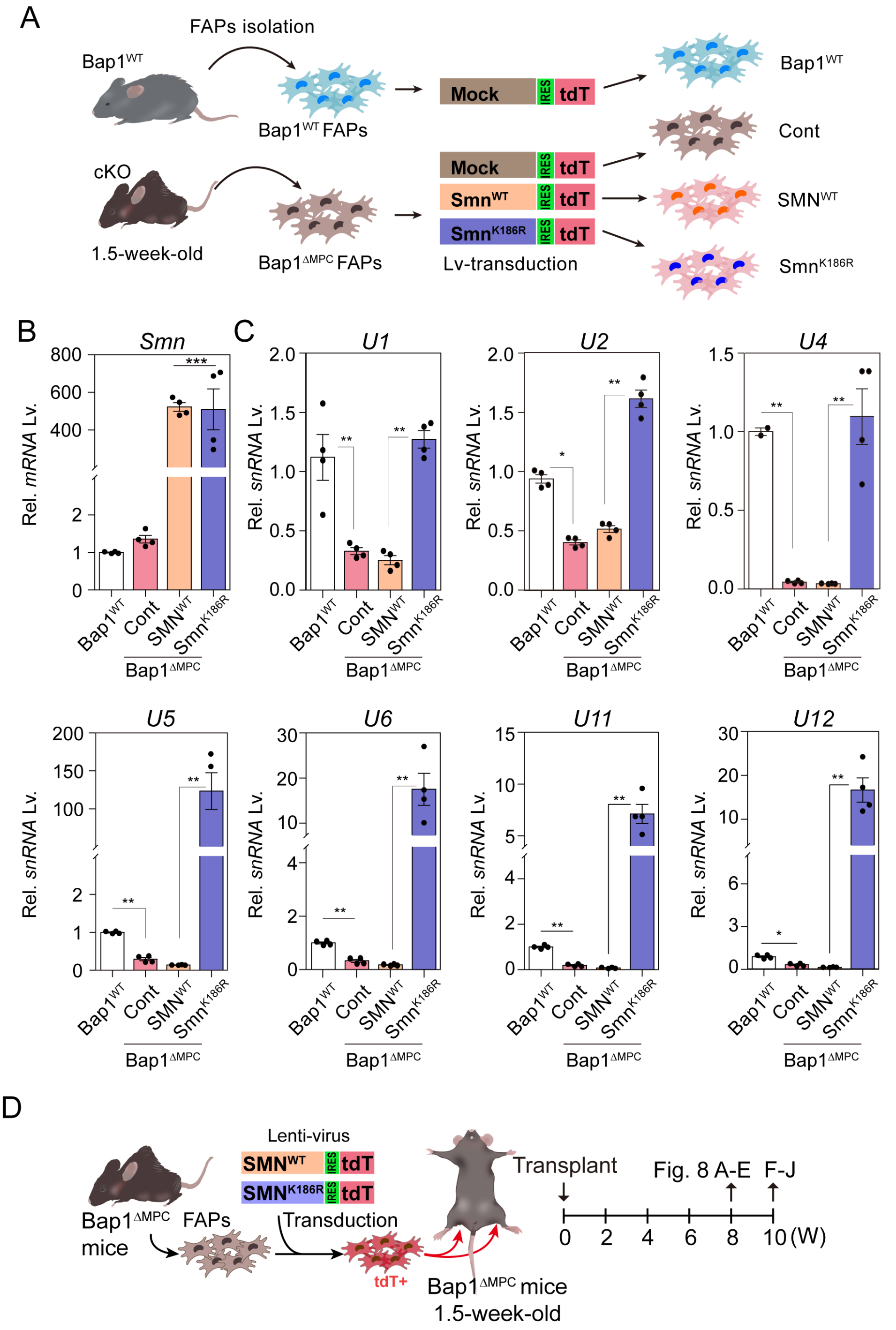

Supplemental Figure 9. Restoration of *snRNA* level by SMN^{K186R} in $Bap1^{\Delta MPC}$ FAPs. (**A**) A scheme for lentiviral transduction. FAPs from hindlimb muscles of 1.5-week-old $Bap1^{WT}$ or $Bap1^{\Delta MPC}$ mice were transduced with lentiviral vectors containing mock-, SMN^{WT}- and SMN^{K186R}-IRES-tdTomato constructs as illustrated. (**B** and **C**) Relative expression of SMN (**B**) and snRNA (**C**) in FAPs 48 hours after transduction. n=4 animals for each group; data are mean±s.e.m.; Mann–Whitney U test; **p*<0.05, ***p*<0.01, ****p*<0.001; n.s, not significant. **(D)** Experimental scheme of FAPs transplantations. $Bap1^{\Delta MPC}$ FAPs from the hindlimb muscles of 1.5-week-old $Bap1^{\Delta MPC}$ mice were transduced with lentiviral vectors containing SMN^{WT}-IRES-tdTomato ($FAPs^{SMNWT}$) or SMN^{K186R}-IRES-tdTomato ($FAPs^{SMNK186R}$). Sixteen hours after

transduction, transduced FAPs were transplanted into TA and GA muscles of 1.5-week-old *Bap1*$^{\Delta MPC}$ mice.

Table S1. The list of primers and antibodies used in this study

Primers

Gene	Primer sequence
Smn qRT F	AAGGCACAGCCAGAAGAAAA
Smn qRT R	TCACAGGTCGGGGAAAGTAG
Bap1 qRT F	TGCCAAATCCCCTATGCAGG
Bap1 qRT R	TTGCTCAACGATCCTGGCTT
Chrna1 qRT F	GTTCTGGGCTCCGAACATGA
Chrna1 qRT R	GATCAGCTGTAGACCCACGG
Chrnb1 qRT F	CTGAATCCGTTTGGCTCCCT
Chrnb1 qRT R	GCACAGAGCCCTCGAAAGAT
Chrng qRT F	TCGTGAACTCTGTGGTCGTG
Chrng qRT R	CGCACATGCATCCGTAACAG
Chrne qRT F	GGATGACGACGGCAATACCA
Chrne qRT R	CCCTCATAGCGGCGAATCAT
U1 qRT F	CCTGGCAGGGGAGATACCATGAT
U1 qRT R	TGCAGTCGAGTTTCCCGCATTT
U2 qRT F	CGGCCTTTTGGCTAAGATCAAGTG
U2 qRT R	TCCTCGGATAGAGGACGTATCAGA
U4 qRT F	GAGGTTTATCCGAGGCGCGATTAT
U4 qRT R	CACGGCGGGGTATTGGGAAAAGTT
U5 qRT F	TTTCCGTGGAGAGGAACAACTCTG
U5 qRT R	CTTGTCAAGACAAGGCCTCAAAA
U6 qRT F	TCGCTTCGGCAGCACATATACT
U6 qRT R	CGCTTCACGAATTTGCGTGTCA
U11 qRT F	CGTGCGGAATCGACATCAAGAGA
U11 qRT R	CAACGATCACCAGCTGCCCAATTA
U12 qRT F	GCCCGAGTCCTCACTGCTTATGT
U12 qRT R	AAAGTAGGCGGGTCGCCTCAGAT
5.8s rRNA qRT F	GCGCTAGCTGCGAGAATTAA
5.8s rRNA qRT R	CAAGTGCGTTCGAAGTGTCG
18s rRNA qRT F	AAACGGCTACCACATCCAAG
18s rRNA qRT R	CCTCCAATGGATCCTCGTTA
Dpp4 qRT F	CACTTTCAGCAGTCAGCTCAG
Dpp4 qRT R	TGTGGGAATAGATGTGCTGGT
Sema3c qRT F	TAGTCTGTCCCACCAGCAGT
Sema3c qRT F	GCCAGCCATTTTGCACTCTT
Pi16 qRT F	GGGGCCACAACAAAGAACG
Pi16 qRT F	CACATCTGGTTCGGATCGCA
Sbsn qRT F	TGGACAGGGGTCTCATCAAG
Sbsn qRT F	CTGGGCATCAGTTTAGGGCA
Efemp1 qRT F	GCGCTGGTCAAGTCACAGTA
Efemp1 qRT F	AAGCATCTGGGACAATGTCAC
Cxcl14 qRT F	GAAGATGGTTATCGTCACCACC
Cxcl14 qRT F	CGTTCCAGGCATTGTACCACT
Smoc2 qRT F	CCCAAGCTCCCCTCAGAAG
Smoc2 qRT F	GCCACACACCTGGACACAT
Col15a1 qRT F	GAGGACTCGGAGCTTTCTGG
Col15a1 qRT F	GCTCCATCCCCTGAACCATC
Sparcl1 qRT F	GCTAGCTCCTCTTGGGCATT
Sparcl1 qRT F	ATGCTGGCTAGATCTGCGG
Bap1 Ex5 (P1; Com.)	TCCCAACTCTTGTGCCACTCA
Bap1 Ex7 (P2; Floxed)	ACGTGGTTCTGGCCTGGAAA
Bap1 Ex13 (P3; Del)	CTGTCCCCTTCCGCTTGTAT
Cre F	GCATTACCGGTCGATGCAACGAGTGATGAG
Cre R	GAGTGAACGAACCTGGTCGAAATCAGTGCG
Rosa Com	AAAGTCGCTCTGAGTTGTTAT
Rosa WT	GCGAAGAGTTTGTCCTCATCC
Rosa MT	GGAGCGGGAGAAATGGATATG

Antibodies

Name	Cat. (clone)	Species	Manufacturer	Conjugate
Anti-Bap1	Sc-28236 (H-300)	Rabbit	Santa Cruz	·
Anti-Bap1	Sc-28383 (C4)	Mouse	Santa Cruz	·
Anti-Smn	610646	Mouse	BD Biosciences	·
Anti-ChAT	AB144P	Goat	Merckmillipore	·
Anti-Neurofilament	AB1987	Rabbit	Merckmillipore	·
Anti-NeuN	MAB377 (A60)	Rabbit	Merckmillipore	·
Anti-Laminin	Ab11576 (4H8-2)	Rat	Abcam	·
Anti-Gapdh	2118 (14C10)	Rabbit	Cell signaling	·
Anti-β-actin	A2066	Rabbit	Sigma aldrich	·
Anti-β-tubulin	2128 (9F3)	Rabbit	Cell signaling	·
Anti-His	Sc-8036 (H3)	Mouse	Santa Cruz	·
Anti-xpress	R910-25	Mouse	Thermo Fisher Scientific	·
Anti-Flag	F1804 (M2)	Mouse	Sigma aldrich	·
Anti-GFP	ab13970	Chicken	Abcam	·
Anti-RFP	PM005	Rabbit	MBL Life Science	·
Anti-HA	Sc-7392(F-7)	Mouse	Santa Cruz	·
Anti-CD45	103111 (30F11)	Mouse	Biolegend	APC
Anti-CD31	102409 (MEC 13.3)	Mouse	Biolegend	APC
Anti-Sca-1	122507 (E13-161.7)	Mouse	Biolegend	FITC
Anti-Vcam1	105703 (429 MVCAM)	Mouse	Biolegend	Biotin
Anti-Dpp4	740021 (H194-112)	Mouse	BD Biosciences	BV421
PE-Cy7 Streptavidin	405206	Mouse	Biolegend	PE-Cy7
HRP-Rabbit IgG	W4011	Rabbit	Promega	HRP
Alexa-488-Rabbit IgG	A11034	Rabbit	Thermo Fisher Scientific	Alexa-488
Alexa-594-Rabbit IgG	A11037	Rabbit	Thermo Fisher Scientific	Alexa-594
HRP-Mouse IgG	W4021	Mouse	Promega	HRP
Alexa-488-Mouse IgG	A11029	Mouse	Thermo Fisher Scientific	Alexa-488
Alexa-594-Mouse IgG	A11032	Mouse	Thermo Fisher Scientific	Alexa-594
Alexa-488-Rat IgG	A11006	Rat	Thermo Fisher Scientific	Alexa-488
Alexa-594-Rat IgG	A11007	Rat	Thermo Fisher Scientific	Alexa-594
Alexa-488-Chicken IgG	A11039	Chicken	Thermo Fisher Scientific	Alexa-488
Alexa-594 Goat IgG	A11058	Goat	Thermo Fisher Scientific	Alexa
Alexa-555-α-Bungarotoxin	B35451	·	Thermo Fisher Scientific	Alexa-555

Supplemental Video 1. Representative video of the movement of 19-month-old *Bap1*$^{\Delta MPC}$ mice.

Supplemental Video 2. Representative video of tail suspension test. YFP^{+} FAPs from 1.5-week-old Prx1-Cre; Rosa-YFP mice were transplanted into 1.5-week-old *Bap1*$^{\Delta MPC}$ mice. Intramuscular injection of PBS into 1.5-week-old *Bap1*$^{\Delta MPC}$ mice was performed as transplantation control. Tail suspension tests were performed 8 weeks after transplantation. Hindlimb clasping in *Bap1*$^{\Delta MPC}$ mice (red arrow) was rescued in FAPs-transplanted *Bap1*$^{\Delta MPC}$ mice (blue arrow).

Supplemental Video 3. Representative video of tail suspension test. FAPs from the hindlimb muscles of 1.5-week-old Rosa-CreER; *Bap1*$^{f/f}$ mice were transplanted into TA and GA muscles of 1.5-week-old *Bap1*$^{\Delta MPC}$ mice. 8weeks after transplantation, the cell-transplanted *Bap1*$^{\Delta MPC}$ mice were orally administered with Tmx for 3 consecutive days**.** Tail suspension tests were performed at the indicated weeks after Tmx administration.

Supplemental Video 4. Representative video of tail suspension test. Dpp4^{+} and Dpp4^{-} FAPs were freshly isolated from 4-week-old tdTomato reporter mice by FACS and were transplanted them into *Bap1*$^{\Delta MPC}$ mice. Intramuscular injection of PBS was performed as transplantation control.

Supplemental Video 5. Representative video of tail suspension test. FAPs from the hindlimb muscles of 1.5-week-old Bap1$^{\Delta MPC}$ mice were transduced with lentiviral vectors containing *SMN*^{WT}-IRES-tdTomato (FAPs^{SMNWT}) or SMN^{K186R}-IRES-tdTomato (FAPs^{SMNK186R}). Sixteen hours after transduction, transduced FAPs were transplanted into TA and GA muscles of 1.5-week-old *Bap1*$^{\Delta MPC}$ mice. Intramuscular injection of PBS was performed as transplantation control. Tail suspension tests were performed 8weeks after transplantation.

References

1. El-Khodor, B.F. *et al.* Identification of a battery of tests for drug candidate evaluation in the SMNDelta7 neonate model of spinal muscular atrophy. *Exp Neurol* **212**, 29-43 (2008).
2. Guyenet, S.J. *et al.* A simple composite phenotype scoring system for evaluating mouse models of cerebellar ataxia. *J Vis Exp* (2010).
3. Hatzipetros, T. *et al.* A Quick Phenotypic Neurological Scoring System for Evaluating Disease Progression in the SOD1-G93A Mouse Model of ALS. *J Vis Exp* (2015).
4. Kim, J.H. *et al.* Sex hormones establish a reserve pool of adult muscle stem cells. *Nat Cell Biol* **18**, 930-940 (2016).
5. Liu, L., Cheung, T.H., Charville, G.W. & Rando, T.A. Isolation of skeletal muscle stem cells by fluorescence-activated cell sorting. *Nat Protoc* **10**, 1612-1624 (2015).
